# A novel base-metal multifunctional catalyst for the synthesis of 2-amino-3-cyano-4*H*-chromenes by a multicomponent tandem oxidation process

**DOI:** 10.1038/s41598-022-06759-7

**Published:** 2022-02-21

**Authors:** Farhad Omarzehi Chahkamali, Sara Sobhani, Jose Miguel Sansano

**Affiliations:** 1 Department of Chemistry, College of Sciences, University of Birjand, Birjand, Iran; 2 Departamento de Química Orgánica, Facultad de Ciencias, Centro de Innovación en Química Avanzada (ORFEO-CINQA), Universidad de Alicante, Apdo. 99, 03080 Alicante, Spain

**Keywords:** Catalysis, Organic chemistry

## Abstract

A novel base-metal multifunctional nanomagnetic catalyst is prepared by the immobilization of tungstate anions onto γ-Fe_2_O_3_ supported with imidazolium moieties. The (γ-Fe_2_O_3_-Im-Py)_2_WO_4_ was fully characterized using FT-IR, XPS, TEM, FESEM, ICP, TGA, VSM and XRD and used as a multifunctional heterogeneous catalyst for the synthesis of 2-amino-3-cyano-4*H*-chromenes via a multicomponent tandem oxidation process starting from alcohols under solvent-free conditions. During this process, tungstate catalyzes the oxidation of a wide range of alcohols in the presence of TBHP as a clean source. The in-situ formed aldehydes are condensed with malononitrile and β-dicarbonyl compounds/naphthols/4-hydroxycumarin through promotion by pyridine and imidazolium moieties of the catalyst. By this method, a variety of 2-amino-3-cyano-4*H*-chromenes are generated in good to high yields from alcohols as inexpensive and easily available starting materials. The catalyst is recovered easily by the aid of an external magnetic field and reused in five successive runs with insignificant decreasing activity.

## Introduction

Tandem reactions, which allow multistep reactions in the same vessel, have attracted an enormous attention as they avoid the separation of intermediates and decrease waste production, thus offering many important economic benefits^1,2^. Within tandem reactions, tandem oxidation process (TOP) has arisen as an inventive tool in the synthesis of organic compounds starting from alcohols^3^. Alcohols are the most environmentally benign chemicals in the organic reactions due to the broad accessibility, low expense, opportunity of being generated from renewable biomass materials, low level of poisoning, and simplicity of usage, storage, transport and dissolving^4^. Pioneering works concerning the growth of the TOP has been done by Robert Ireland during his attempts for the synthesis of polyether ionophore antibiotics^5^. A number of volatile aldehydes were produced which made their separation difficult. Ireland solved this problem by Swern oxidation of alcohols and subsequent Wittig reagent addition to in-situ generated aldehydes. Since this discovery, a diversity of worthwhile materials has been synthesized from alcohols by TOP^6–8^. Recently, TOP has expanded concerning the use of a variety of oxidizing agents and nucleophilic compounds^9–13^. More importantly, expansion of TOP to multicomponent reactions (MCRs) has offered the synthesis of a wide range of novel valuable compounds^14–18^. These processes provide high atom economy, and diminish the number of time-loosing, cost-demanding, waste-generating operations and purification processes in the chemical synthesis^19^. In the past few years many attentions have been paid to the development and application of multifunctional catalysts by placing different types of active sites on one catalyst for using in tandem catalysis process^20–22^. However, the application of multifunctional catalytic systems in the multicomponent TOP starting from alcohols is a new field of studies^23–25^.

Chromene containing compounds are a very important family of heterocycles with wide biological properties and therapeutic applications^26–28^. They exist in the widespread natural compounds and display numerous pharmaceutical properties such as anti-inflammatory, anti-oxidant, anti-bacterial, anti-cancer, anti-coagulant, anti-microbial, anti-Alzheimer and anti-HIV^29,30^. 2-Amino-3-cyano-4*H*-chromenes are generally prepared by three-component reactions involving the cyclocondensation of various types of aldehydes, malonates and β-dicarbonyl compounds or activated phenols^31^. Numerous modified catalysts have been used for the elaboration of 2-amino-3-cyano-4*H*-chromenes and their derivatives^32–37^. Most of the reports suffer from drawbacks e.g., long reaction times, difficult workup procedures, use of unrecyclable catalyst and afford only moderate yields of the products. 2-Amino-3-cyano-4*H*-chromenes can also be prepared from alcohols via multicomponent TOP reactions. This method passes through three sequential steps: (1) oxidation of alcohols to aldehydes which requires an oxidizing catalyst, (2) Knoevenagel condensation of the in-situ formed aldehydes with malononitrile, (3) Michael addition of β-dicarbonyl compounds followed by cyclization reaction. The last two steps can be promoted by acidic and/or basic catalysts. Based on a literature survey, there is a few reports on the preparation of 2-amino-3-cyano-4*H*-chromene starting from alcohol^38–41^. Within these reports, there is only one report on using a bifunctional catalyst for the preparation of these targeted scaffolds^41^. These methods suffered from several drawbacks such as using expensive oxidizing agent, requiring pH adjustment, time-consuming catalyst isolation, limited activated nucleophiles/alcohols and long reaction time.

The high selective and controllable oxidation of alcohols to corresponding aldehydes is one of the predominant and challenging reactions in synthetic chemistry^42,43^. The classical oxidation methods include the use of stoichiometric amounts of strong oxidants such as chromium (VI) or manganese (VII) reagents^44,45^ and concentrated HNO_3_^46^ which are environmentally hazardous and produce large amounts of toxic wastes. A green protocol to replace the classical method for oxidation reaction is using oxidation metal catalysts^47–49^. However, while various catalytic systems including molybdenum^50^, manganese^51^, iron^52^, palladium^53^, rhenium^54^, ruthenium^55^, and copper^56^, have been well explored, catalytic systems based on tungsten have been particularly received a great deal of attentions to achieve high efficiency and selectivity in the oxidation of alcohols^57,58^. As there is a difference in solubility of tungstate anion and organic substrates, the use of ionic liquids containing tungstate anions has attracted much attention for the oxidation of alcohols due to the unique property of ionic liquids (ILs) as a phase transfer catalyst under organic–inorganic media^59–62^. On the other hand, immobilization of tungsten species onto solid supports has been evolving to overcome the difficult separation of catalyst and products under similar homogeneous conditions. Along this line, several heterogeneous imidazolium-based ILs containing tungstate anions have been introduced for the selective oxidation of alcohols^63–65^.

**Figure 1:**
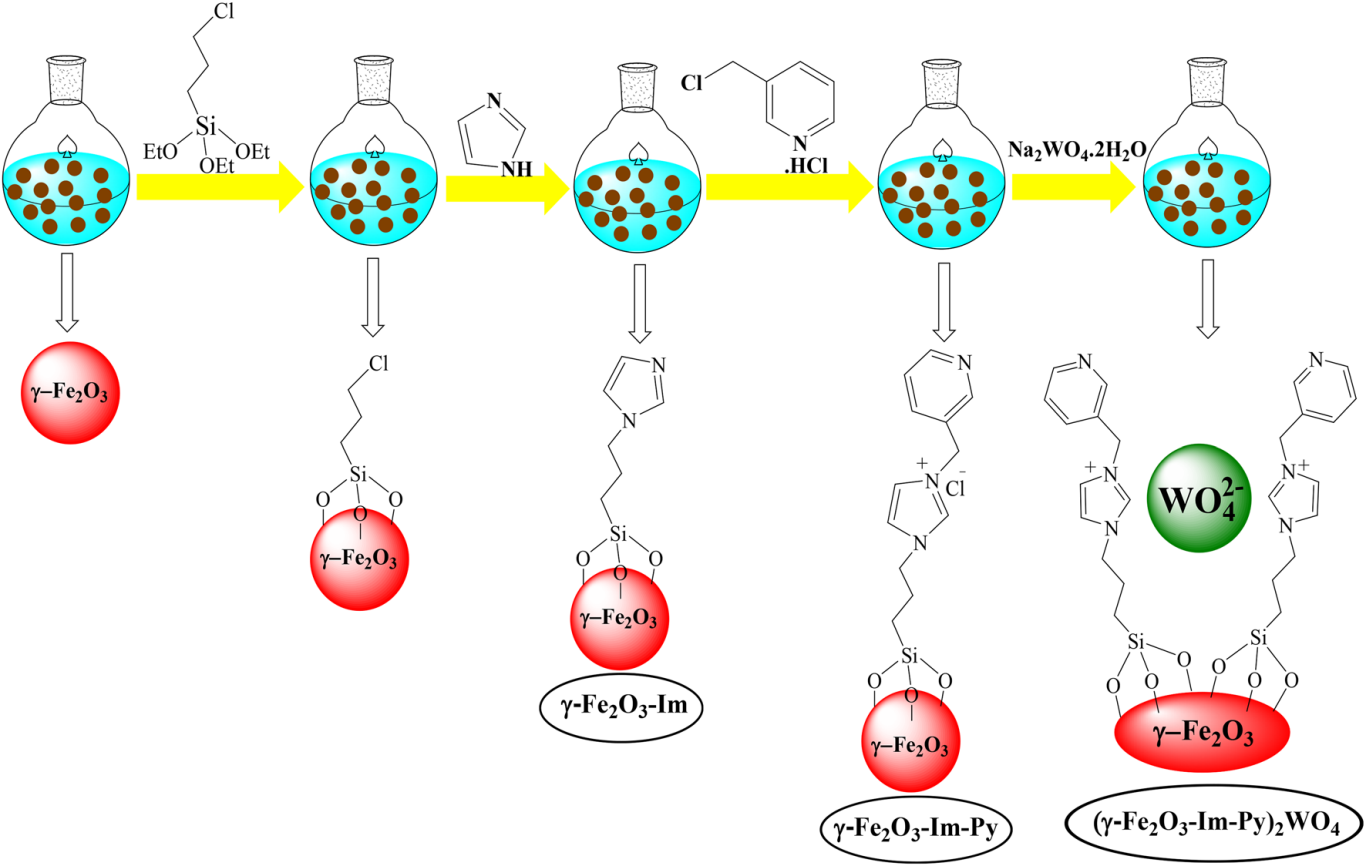
Synthesis of multifunctional (γ-Fe_2_O_3_-Im-Py)_2_WO_4_.

Recently, we have prepared two new magnetically separable functionalized Pd–*N*-heterocyclic carbene (NHC) starting from supported imidazolium salts on magnetic iron oxide (MNPs) and reported their applications in several organic transformations^66,67^. In our reported catalysts, we have profited from the presence of two nitrogen atoms in imidazole for the immobilization of imidazole onto a MNPs as a solid support on one side and then functionalization of supported imidazolium salts on the other side. Following our attempts for the developing multifunctional heterogeneous catalysts in organic reactions^68–70^, herein, we have tried to design a new base-metal multifunctional catalyst from supported imidazole onto MNPs for the generation of 2-amino-3-cyano-4*H*-chromenes via a three-component TOP starting from alcohols. For this purpose, at first the free nitrogen of supported imidazole was functionalized with pyridine by the reaction with 3-(chloromethyl) pyridine hydrochloride and then the chloride anion in the resulting imidazolium ILs was exchanged with tungstate to produce (γ-Fe_2_O_3_-Im-Py)_2_WO_4_ (Fig. 1). In this catalyst, we predicted that tungstate anions will promote the selective oxidation of alcohols to aldehydes and pyridine will activate Knoevenagel condensation-Michael addition-cyclization reaction of in-situ formed aldehydes with malononitrile and dimedone.

## Experimental

### General information

All Chemicals and solvents were bought from Merck Chemical Company. The product purity and the reaction progress were investigated by TLC using silica gel polygram SILG/UV254 plates. The Fourier transform infrared (FT-IR) spectra were recorded on a Shimadzu Fourier Transform Infrared Spectrophotometer (FT-IR-8300). X-ray photoelectron spectroscopy (XPS) analyses were accomplished using a VG-Microtech Multilab 3000 spectrometer, equipped with an Al anode. The deconvolution of spectra was performed by using Gaussian Lorentzian curves. The content of W in the catalyst was determined by OPTIMA 5300DV ICP analyzer. The transmission electron microscopy (TEM) analysis was performed using Philips EM208S operating at 100 kV. FESEM were achieved using a TESCAN MIRA3. Thermo-gravimetric analysis (TGA) was carried out using TA-Q600. The vibrating sample magnetometer (VSM) analysis was performed using Lake Shore Cryotronics 7407. X-ray diffraction (XRD) analysis was performed using XRD Philips PW1730. Melting points were measured by an electrothermal 9100 apparatus.

### Synthesis of chloro-functionalized γ-Fe_2_O_3_^71^

At first, γ-Fe_2_O_3_ (2.0 g) was dispersed in dry toluene (40 mL) by sonication for 45 min. 3-Chloropropyl triethoxysilane (3 mL) was slowly added and stirred while it was heated to 105 °C. Then, the stirring was continued for 48 h at the same temperature. Chloro-functionalized γ-Fe_2_O_3_ was obtained after separation of the solid using an external magnetic field, washing with diethyl ether and dichloromethane, and vacuum-dried.

### Synthesis of imidazole supported on γ-Fe_2_O_3_ (γ-Fe_2_O_3_-Im)^66^

Chloro-functionalized γ-Fe_2_O_3_ (1.6 g) was dispersed in toluene (30 mL, dry) by sonication (30 min). While the mixture was stirred, imidazole (0.204 g, 3 mmol) was added and then refluxed at 110 °C. After 24 h, Et_3_N (0.43 mL, 3 mmol) was added to the cooled mixture and stirred (30 min). The solid material was isolated using an external magnet, washed with water (3 × 10 mL) and acetone (3 × 10 mL) and dried in a vacuum oven (70 °C).

### Synthesis of γ-Fe_2_O_3_-Im-Py

The synthesized γ-Fe_2_O_3_-Im (1.5 g) was dispersed in toluene (30 mL, dry) by sonication (30 min). 3-(Chloromethyl) pyridine hydrochloride (0.492 g, 3 mmol) and Et_3_N (0.43 mL, 3 mmol) was added and refluxed at 110 °C. After 24 h, the solid was isolated by an external magnet, washed with H_2_O (3 × 10 mL), EtOH (2 × 10 mL) and acetone (2 × 10 mL) and dried in a vacuum oven (70 °C).

### Synthesis of (γ-Fe_2_O_3_-Im-Py)_2_WO_4_

The synthesized γ-Fe_2_O_3_-Im-Py (1 g) was dispersed in H_2_O (25 mL, deionized) by sonication (30 min). Na_2_WO_4_·2H_2_O (1.319 g, 4 mmol) was added and stirred at ambient temperature. After 48 h, the resulting compound was isolated using an external magnet and washed with H_2_O (3 × 10 mL) and ethanol (2 × 10 mL) to eliminate the unreacted Na_2_WO_4_·2H_2_O and dried in a vacuum oven (70 °C).

### Synthesis of (γ-Fe_2_O_3_-Im-Py)_2_MoO_4_

The synthesized γ-Fe_2_O_3_-Im-Py (1 g) was dispersed in H_2_O (25 mL, deionized) by sonication (30 min). Na_2_MoO_4_.2H_2_O (0.968 g, 4 mmol) was added and stirred at ambient temperature. After 48 h, the resulting compound was isolated using an external magnet and washed with H_2_O (3 × 10 mL) and ethanol (2 × 10 mL) to eliminate the unreacted Na_2_MoO_4_·2H_2_O and dried in a vacuum oven (70 °C). ICP analysis of the resulting (γ-Fe_2_O_3_-Im-Py)_2_MoO_4_ indicated that 0.31 mmol (0.0297 mg) molybdate was immobilized on 1 gr of this compound.

### Synthesis of γ-Fe_2_O_3_-Im-Py-VO_3_

The synthesized γ-Fe_2_O_3_-Im-Py (1 g) was dispersed in H_2_O (25 mL, deionized) by sonication (30 min). NaVO_3_ (0.487 g, 4 mmol) was added and stirred at ambient temperature. After 48 h, the resulting compound was isolated using an external magnet and washed with H_2_O (3 × 10 mL) and ethanol (2 × 10 mL) to eliminate the unreacted NaVO_3_ and dried in a vacuum oven (70 °C). ICP analysis of the resulting γ-Fe_2_O_3_-Im-Py-VO_3_ indicated that 0.64 mmol (0.0326 mg) vanadate was immobilized on 1 gr of this compound.

### Synthesis of γ-Fe_2_O_3_-Im-Me^66^

γ-Fe_2_O_3_-Im (1 g) was dispersed in toluene (30 mL, dry) by sonication (30 min). Methyl iodide (0.25 mL, 4 mmol) was added and stirred under reflux conditions at 110 °C. After 24 h, the solid was collected using an external magnet, washed with diethyl ether (3 × 10 mL) and acetone (3 × 10 mL) and dried in a vacuum oven (70 °C).

### Synthesis of (γ-Fe_2_O_3_-Im-Me)_2_WO_4_

The synthesized γ-Fe_2_O_3_-Im-Me (1 g) was dispersed in H_2_O (25 mL, deionized) by sonication (30 min). Na_2_WO_4_·2H_2_O (1.319 g, 4 mmol) was added and stirred at room temperature. After 48 h, by means of an external magnet, the resulting compound was isolated and washed with H_2_O (3 × 10 mL) and ethanol (2 × 10 mL) to eliminate the unreacted Na_2_WO_4_·2H_2_O and dried in a vacuum oven (70 °C).

### Catalytic performance of (γ-Fe_2_O_3_-Im-Py)_2_WO_4_

#### General procedure for the oxidation of alcohols

A mixture of alcohol (1 mmol), *tert*-butyl hydroperoxide (TBHP) (6 mmol) and (γ-Fe_2_O_3_-Im-Py)_2_WO_4_ (2 mol%, 57 mg) was stirred under solvent-free conditions at 90 °C. The progress of the reaction was monitored by TLC. After requisite time (Table 2), the reaction mixture was cooled to ambient temperature. EtOAc (5 mL) was added to the reaction mixture. (γ-Fe_2_O_3_-Im-Py)_2_WO_4_ was isolated using an external magnet, washed with EtOAc (2 × 5 mL) and EtOH (2 × 5 mL), vacuum dried, and recycled for the next run. The combined organic layer was then dried using Na_2_SO_4_. After solvent evaporation, a crude product was obtained. The pure product was achieved by column chromatography on silica gel eluting *n*-hexane/EtOAc (10:2).

#### General procedure for the three-component TOP synthesis of 2-amino-3-cyano-4***H***-chromene catalyzed by (γ-Fe_2_O_3_-Im-Py)_2_WO_4_

In a round-bottomed flask, alcohol (1 mmol), *tert*-butyl hydroperoxide (TBHP) (6 mmol) and (γ-Fe_2_O_3_-Im-Py)_2_WO_4_ (5 mol%, 142 mg) were mixed and stirred at 90 °C for a defined time (Table 4, Figs. 10 and 11). Then, malononitrile (1.2 mmol) and β-dicarbonyl compounds/naphthols/4-hydroxycumarin (1.2 mmol) was added and stirred at 90 °C for an appropriate time (Table 4, Figs. 10 and 11). After cooling the reaction mixture to ambient temperature, EtOAc (10 mL) was added and the catalyst was collected by an external magnet. It was washed with EtOAc (2 × 5 mL), EtOH (2 × 5 mL), dried and reused for the next run under the same reaction conditions. The combined organic solvents were vacuum evaporated to produce a crude product. Column chromatography on SiO_2_ eluting with *n*-hexane/EtOAc (7:3) produces the pure product. The products were characterized by ^1^H NMR spectra (Supplementary Figs. S1–S14).

## Results and discussion

### Synthesis and characterization of the multifunctional (γ-Fe_2_O_3_-Im-Py)_2_WO_4_

**Figure 2:**
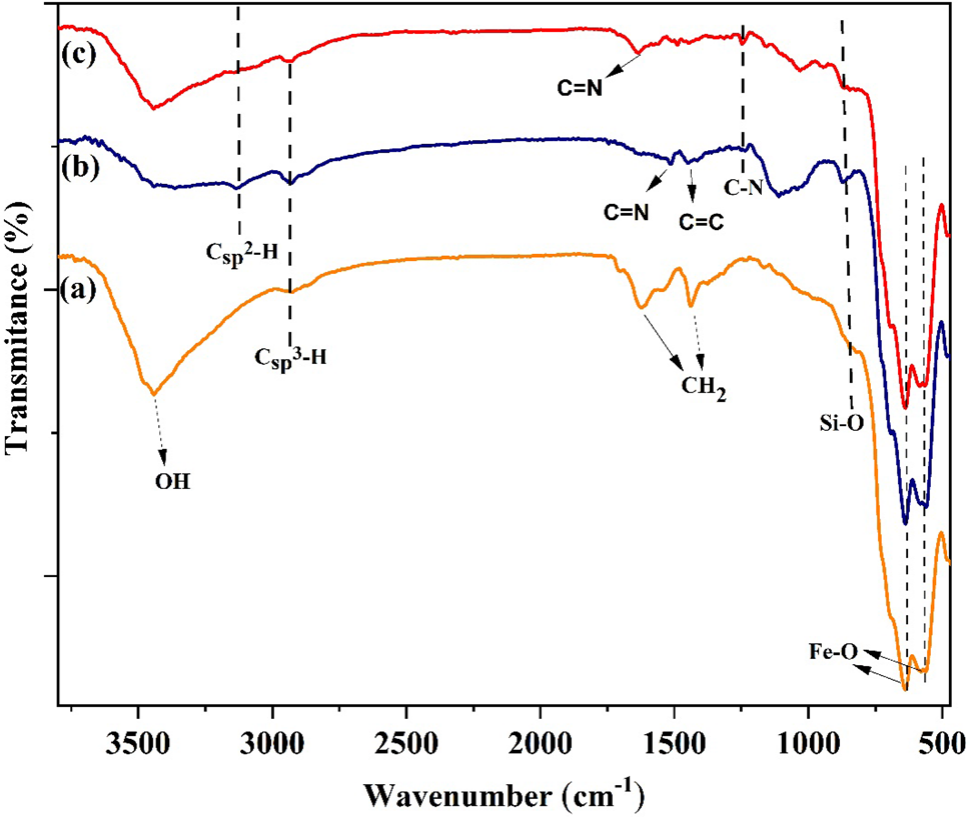
FT-IR spectra of (**a**) chloro-functionalized-γ-Fe_2_O_3_, (**b**) γ-Fe_2_O_3_-Im and (**c**) (γ-Fe_2_O_3_-Im-Py)_2_WO_4_.

We have prepared (γ-Fe_2_O_3_-Im-Py)_2_WO_4_ following the steps designated in Fig. 1. In the first step, γ-Fe_2_O_3_ was functionalized by the reaction with 3-chloropropyltriethoxysilane. Then, the reaction of chloro-functionalized-γ-Fe_2_O_3_ with imidazole led to the formation of γ-Fe_2_O_3_-Im. Imidazole moiety in the γ-Fe_2_O_3_-Im was functionalized by the reaction with 3-(chloromethyl) pyridine hydrochloride to give γ-Fe_2_O_3_-Im-Py. Finally, (γ-Fe_2_O_3_-Im-Py)_2_WO_4_ was prepared by mixing γ-Fe_2_O_3_-Im-Py with Na_2_WO_4_.2H_2_O. The synthesized (γ-Fe_2_O_3_-Im-Py)_2_WO_4_ was characterized by a variety of techniques such as FT-IR, XPS, TEM, FESEM, ICP, VSM, TGA and XRD. The FT-IR spectra of chloro-functionalized-γ-Fe_2_O_3_, γ-Fe_2_O_3_-Im and (γ-Fe_2_O_3_-Im-Py)_2_WO_4_ are presented in Fig. 2. The absorption bands at about 560–640, 875 and 2935 cm^−1^ were related to the stretching vibrations of the Fe–O, Si–O and Csp^3^–H bonds, respectively. In the spectrum of γ-Fe_2_O_3_-Im and (γ-Fe_2_O_3_-Im-Py)_2_WO_4_, the absorption bands at around 1250 and 3135 cm^−1^ were allocated to the stretching vibration of C–N and Csp^2^–H bonds. Peaks appeared at 1530 and 1430 cm^−1^ in the FT-IR of γ-Fe_2_O_3_-Im were assigned to the stretching vibration of C=N and C=C bonds in the imidazole. These absorption bands occurred at around 1630 and 1440–1490 cm^−1^ in the FT-IR spectrum of (γ-Fe_2_O_3_-Im-Py)_2_WO_4_, and confirmed the presence of both imidazole and pyridine anchored on the surface.

**Figure 3:**
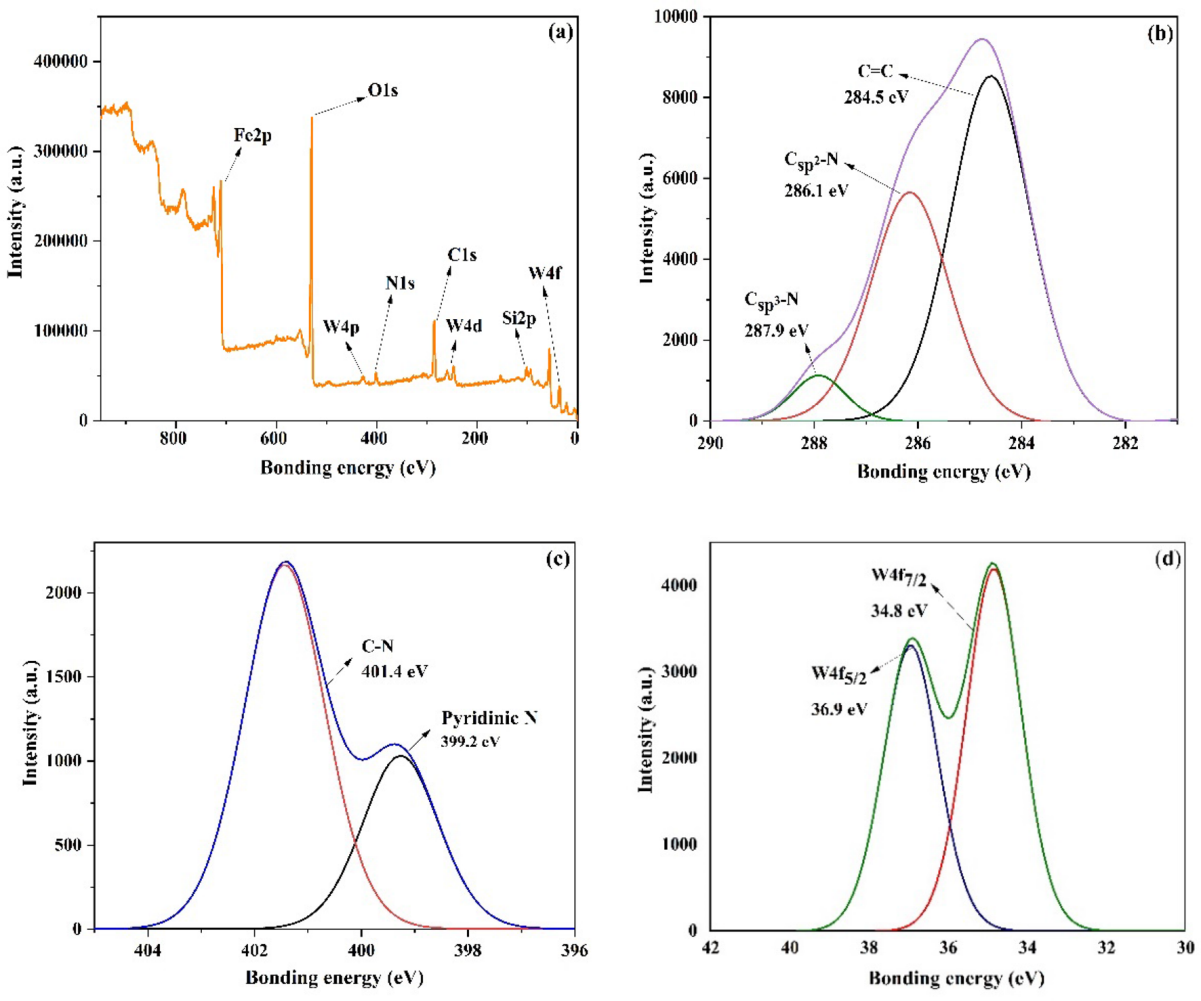
XPS spectra of (**a**) (γ-Fe_2_O_3_-Im-Py)_2_WO_4_, (**b**) C1s, (**c**) N1s and (**d**) W4f.

XPS spectrum of (γ-Fe_2_O_3_-Im-Py)_2_WO_4_ and the detailed XPS spectra for each element are presented in Fig. 3. XPS discovered C1s, O1s, N1s, Fe2p, Si2p, W4p, W4d and W4f states in (γ-Fe_2_O_3_-Im-Py)_2_WO_4_ (Fig. 3a). In Fig. 3b, high-resolution C1s spectra of the catalyst has three main peaks at 284.5 (C–C and C=C), 286.1 (Csp^2^–N) and 287.9 eV (Csp^3^–N)^72^. The N1s spectrum exhibited two main peaks, revealing the presence of the pyridinic nitrogen (399.2 eV) and C–N (401.4 eV) (Fig. 3c)^73^. The XPS spectra of W4f (Fig. 3d) showed two peaks centered at 34.8 and 36.9 eV, related to W^6+^ (W4f_7/2_, W4f_5/2_)^74^.

**Figure 4:**
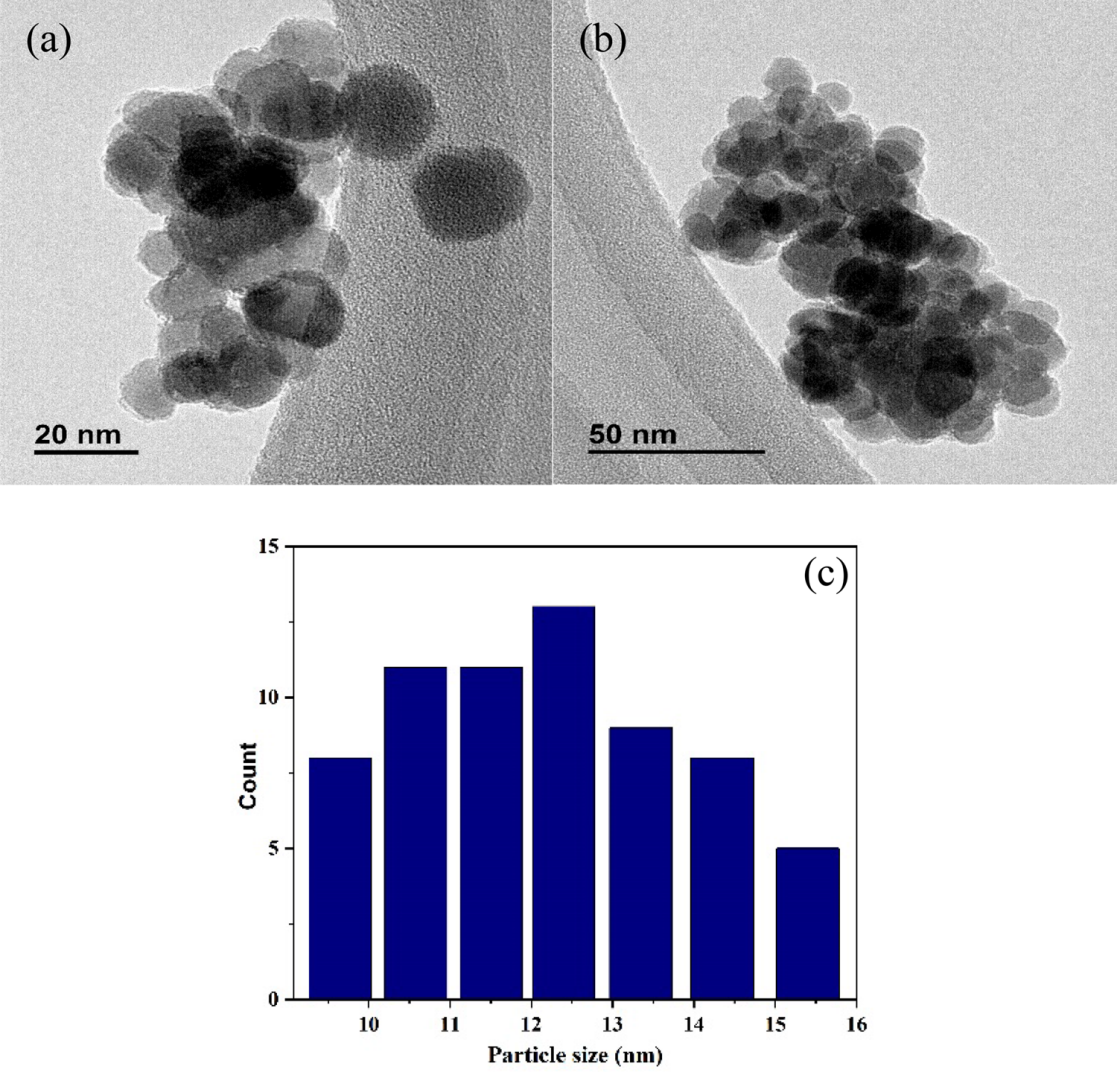
(**a**,**b**) TEM images and (**c**) particle size distribution histogram of (γ-Fe_2_O_3_-Im-Py)_2_WO_4_.

**Figure 5:**
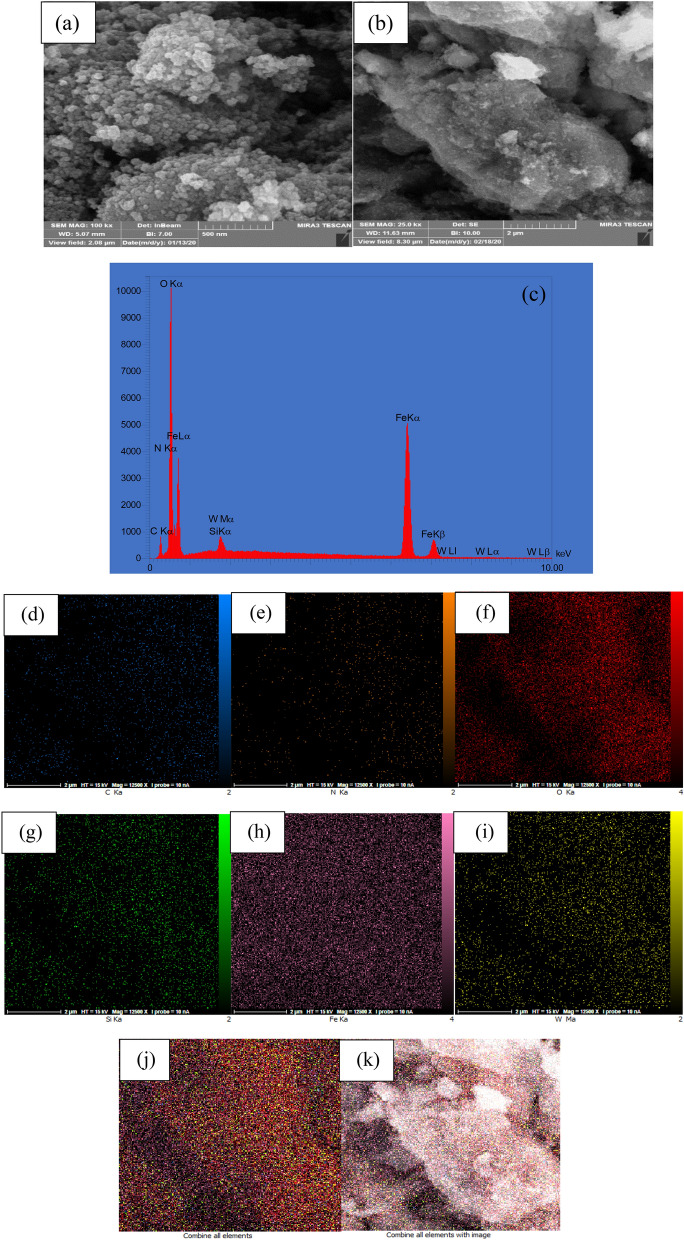
(**a**,**b**) FESEM images of (γ-Fe_2_O_3_-Im-Py)_2_WO_4_, (**c**) EDX spectrum and the corresponding quantitative EDS element mapping of (**d**) C, (**e**) N, (**f**) O, (**g**) Si, (**h**) Fe, (**i**) W and (**j**,**k**) all elements.

ICP analysis of (γ-Fe_2_O_3_-Im-Py)_2_WO_4_ indicated that 0.35 mmol (0.064 mg) tungstate was immobilized on 1 gr of this compound. The size and morphology of (γ-Fe_2_O_3_-Im-Py)_2_WO_4_ were studied using TEM (Fig. 4) and FESEM (Fig. 5). The TEM and FESEM images of (γ-Fe_2_O_3_-Im-Py)_2_WO_4_ exhibit the development of uniform sphere-shaped nanoparticles. The average particle size of (γ-Fe_2_O_3_-Im-Py)_2_WO_4_ was assessed as 12.4 nm using a size distribution histogram (Fig. 4c). Energy-dispersive X-ray spectroscopy (EDS) was done to approve the presence of each element in this compound. The EDS spectrum (Fig. 5c) displays characteristic signals referring to carbon, nitrogen, oxygen, silicon, iron and tungsten, which shows the immobilizing of WO_4_-Im-Py on the surface of the MNPs. Moreover, elemental mapping was performed to realize the spreading of the elements present in the (γ-Fe_2_O_3_-Im-Py)_2_WO_4_. The elemental mapping images (Fig. 5d–k) reveal uniform distribution of all the elements.

**Figure 6:**
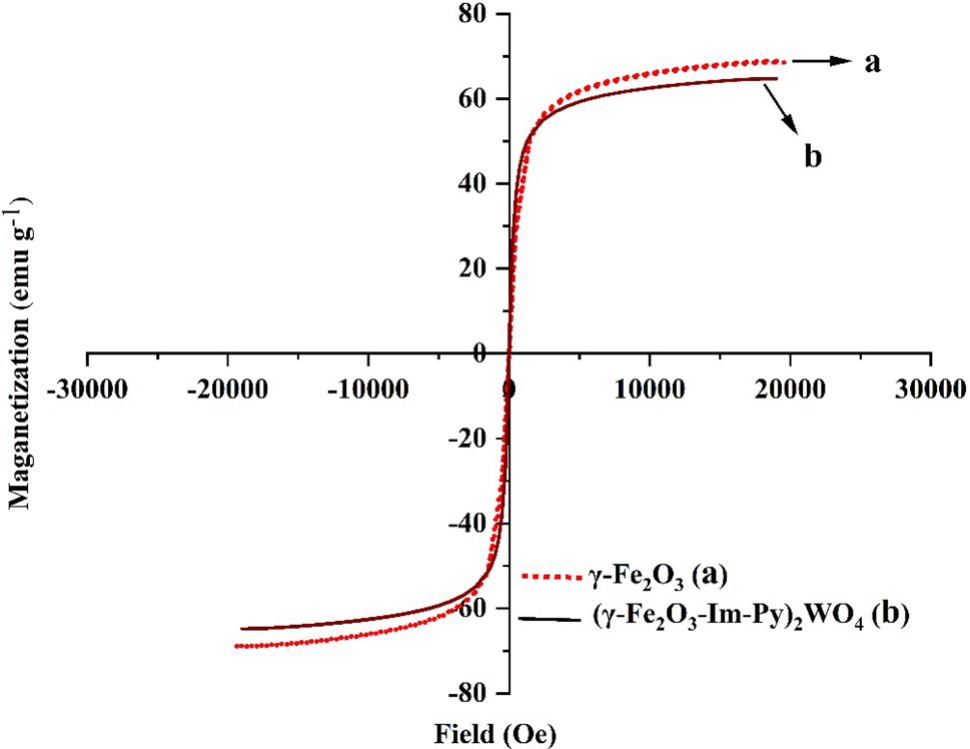
Magnetization curves of (**a**) γ-Fe_2_O_3_ and (**b**) (γ-Fe_2_O_3_-Im-Py)_2_WO_4_.

The magnetization curves of γ-Fe_2_O_3_ and (γ-Fe_2_O_3_-Im-Py)_2_WO_4_ were shown in Fig. 6. The magnetizations of γ-Fe_2_O_3_ and (γ-Fe_2_O_3_-Im-Py)_2_WO_4_ are 68.5 and 64.8 emu/g, respectively. These results indicate the paramagnetic nature of the catalyst. Because of the coating of MNPs, the magnetization value of the catalyst is faintly lesser than that of γ-Fe_2_O_3_. This low decrease in the magnetization does not affect the catalyst isolation from the reaction.

**Figure 7:**
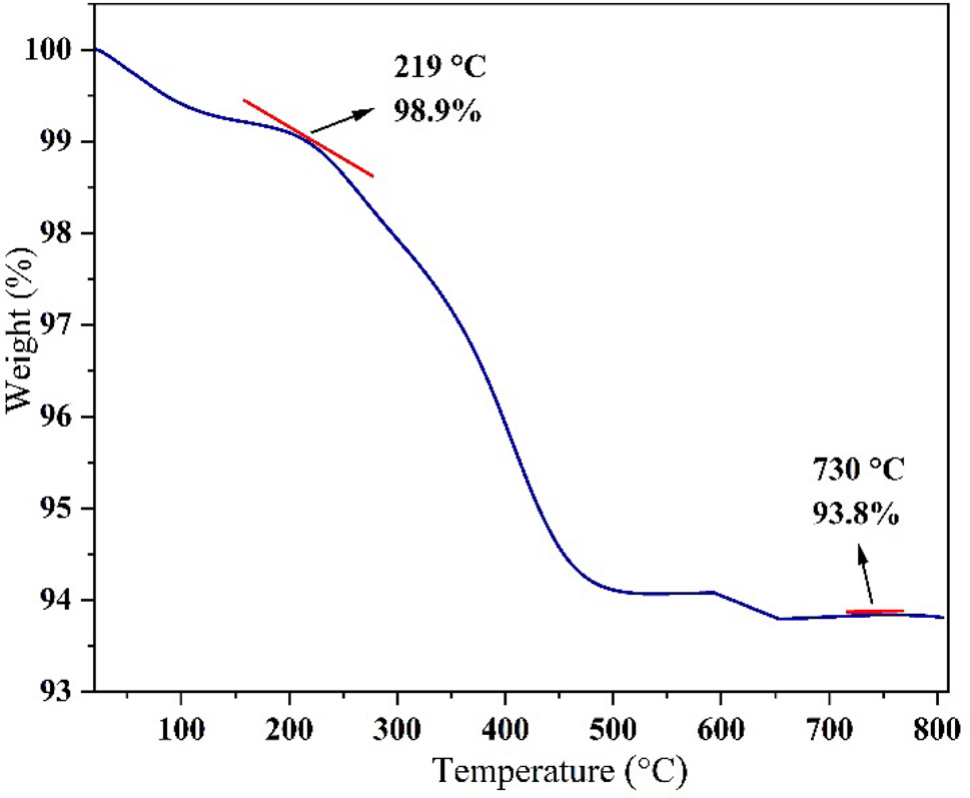
TGA of (γ-Fe_2_O_3_-Im-Py)_2_WO_4_.

Thermal stability of (γ-Fe_2_O_3_-Im-Py)_2_WO_4_ was analyzed by thermogravimetry (TGA) under inert atmosphere (nitrogen) using 10 °C/min heating slope in the range of 20 to 810 °C. TGA curve of (γ-Fe_2_O_3_-Im-Py)_2_WO_4_ (Fig. 7) demonstrates the two-step compound decomposition. A 1.1% weight loss can be observed below 219 °C, which is due to removal of physically adsorbed water molecules. In the second stage, 5.1% mass loss can be observed at 219–730 °C due to the decomposition of organic species supported on the MNPs surface. TGA curve also approves the fruitful loading of organic moiety on the surface of MNPs.

**Figure 8:**
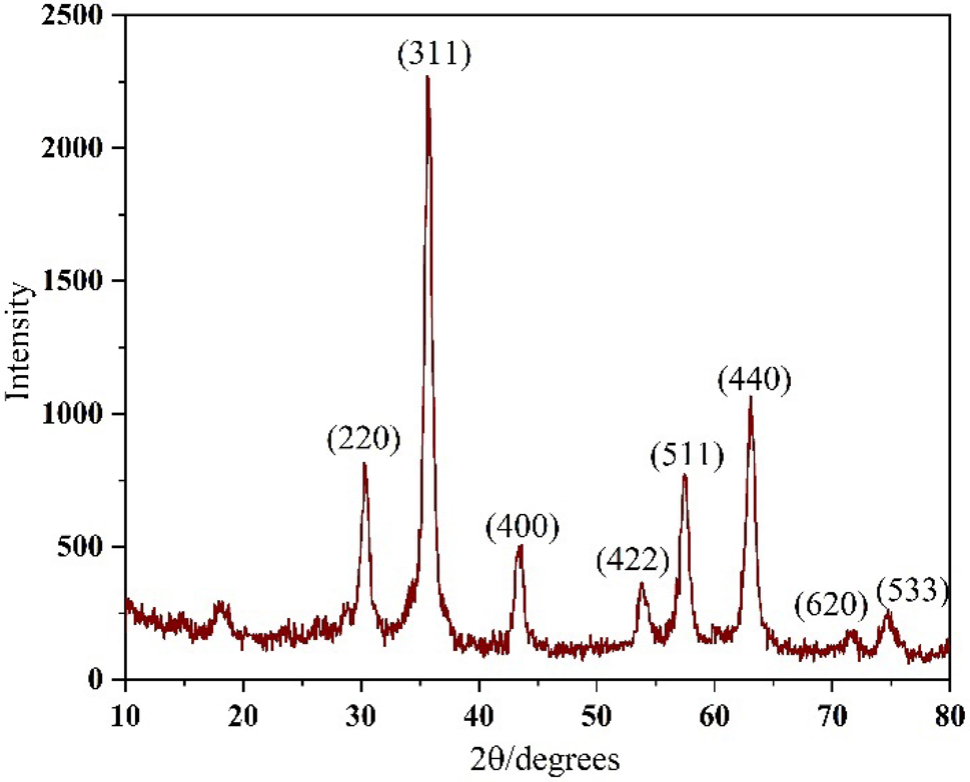
XRD pattern of (γ-Fe_2_O_3_-Im-Py)_2_WO_4_.

Figure 8 displays the XRD patterns of the (γ-Fe_2_O_3_-Im-Py)_2_WO_4_. The diffraction peaks positioned at 30.44°, 35.75°, 43.5°, 53.9°, 57.4°, 62.85°, 71.75° and 74.6°, which are indexed to (220), (311), (400), (422), (511), (440), (620) and (533), respectively. Their relative intensities match pretty well with the inverse spinal structure of maghemite according to JCPDS card No. 39-1346^75^. These observations confirmed the existence of γ-Fe_2_O_3_ nanocrystals and indicates that the crystalline phase of γ-Fe_2_O_3_ did not alter in the stages of the catalyst synthesis.

### Investigation of catalytic activity of the (γ-Fe_2_O_3_-Im-Py)_2_WO_4_ in the synthesis of 2-amino-3-cyano-4*H*-chromenes via multicomponent TOP

We have firstly investigated the activity of the catalyst for the oxidation of alcohols by choosing benzyl alcohol as a model compound. The influence of diverse oxidants, solvents, temperatures and amounts of the catalyst and the oxidant was examined in the oxidation reaction of benzyl alcohol to benzaldehyde (Table 1). The best result was accomplished using 2 mol% of the catalyst and 6 equivalents of TBHP under solvent-free conditions at 90 °C.

**Table 1:** Optimization of reaction conditions in the oxidation of benzyl alcohol catalyzed by (γ-Fe_2_O_3_-Im-Py)_2_WO_4_.

Entry	Catalyst^a^ (mol%)	Oxidant (equiv.)	Solvent	Temperature (°C)	Time (h)	Yield^b^ (%)
1	1.5	H_2_O_2_ (6)	H_2_O	90	5	65
2	1.5	TBHP (6)	H_2_O	90	6	79
3	1.5	Oxane (6)	H_2_O	90	8	50
4	1.5	Air	H_2_O	90	24	55
5	1.5	O_2_ (balloon)	H_2_O	90	10	68
6	1.5	TBHP (6)	–	90	4	87
7	1.5	TBHP (6)	CH_3_CN	–^c^	5	80
8	1.5	TBHP (6)	EtOH	–^c^	7	62
9	2	TBHP (6)	–	90	3	98
10	1	TBHP (6)	–	90	10	75
11	2	TBHP (6)	–	70	5	83
12	2	TBHP (6)	–	50	8	55
13	2	TBHP (6)	–	r.t	24	40
14	2	TBHP (4)	–	90	5	80
15	2	TBHP (2)	–	90	10	45
16	2	–	–	90	24	15

^a^Based on W content. ^b^Isolated yield. Reaction conditions: benzyl alcohol (1 mmol), solvent (3 mL). ^c^Reflux.

To test the generality of this protocol, a diversity of primary and secondary alcohols was allowed to be oxidized under obtained optimum reaction conditions. As shown in Table 2, benzyl alcohols (primary and secondary) were selectively oxidized to aldehydes or ketones in good to high yields (entries 1–18) without any overoxidation to carboxylic acid or ester. Furfuryl alcohol, as a well-known challenging heteroaromatic alcohol, was oxidized selectively to furfural (Table 2, entry 19). Aliphatic alcohols represented lower efficiency under similar oxidation reaction conditions than benzyl alcohols (Table 2, entries 20–22).

**Table 2:** Oxidation of alcohols to carbonyl compounds with TBHP catalyzed by (γ-Fe_2_O_3_-Im-Py)_2_WO_4_.

Entry	Alcohol	Product	Time (h)	Yield^a^ (%)
1			3	98
2			5	85
3			5	87
4			3.5	95
5			4	93
6			4	93
7			3.5	92
8			3	95
9			4	88
10			5	83
11			4	82
12			5	85
13			4	87
14			5	94
15			5	91
16			6	90
17			6	88
18			7	82
19			4	91
20			12	55
21			13	46
22			15	40

^a^Isolated yield. Reaction conditions: benzyl alcohol (1 mmol), TBHP (6 mmol) and (γ-Fe_2_O_3_-Im-Py)_2_WO_4_ (57 mg, 2 mol%: based on W content and relative to alcohol) at 90 °C.

**Figure 9:**
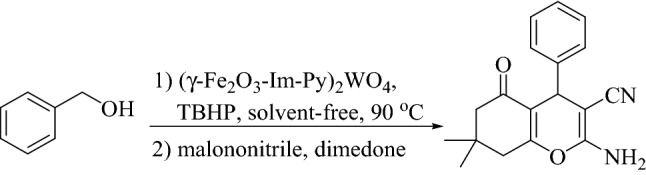
Synthesis of 2-amino-3-cyano-4*H*-chromenes via multicomponent TOP from benzyl alcohol using different amounts of the catalyst.

With successful alcohol oxidation, we studied the utility of (γ-Fe_2_O_3_-Im-Py)_2_WO_4_ in the synthesis of 2-amino-3-cyano-4*H*-chromenes via a multicomponent tandem oxidation process (Fig. 9). Thus, the reaction between benzyl alcohol, malononitrile and dimedone was selected as a model reaction to find the optimum amount of the catalyst (Table 3, entries 1–4). The best result was reached using 5 mol% of the catalyst (Table 3, entry 4).

**Table 3:** Synthesis of 2-amino-3-cyano-4*H*-chromenes via multicomponent TOP using different amounts of the catalyst.

Entry	Catalyst^a^ (mol%)	Time^b^ (I + II, h)	Isolated yields (%)
1	2	3 + 7	54
2	3	2 + 7	68
3	4	2 + 5	80
4	5	1.5 + 4	91

^a^Amount of the catalyst is based on W content and relative to alcohol. ^b^Reaction conditions: (I) benzyl alcohol (1 mmol), TBHP (6 mmol), (γ-Fe_2_O_3_-Im-Py)_2_WO_4_, neat, 90 °C; and (II) malononitrile (1.2 mmol), dimedone (1.2 mmol), neat, 90 °C.

**Figure 10:**
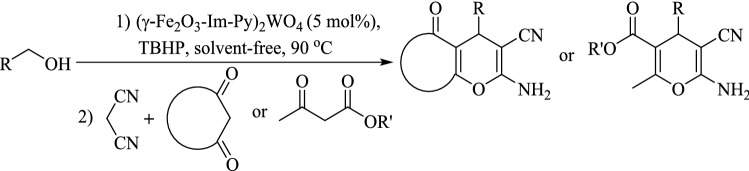
Reactions of a variety of aromatic/aliphatic alcohol, malononitrile and β-dicarbonyl compounds.

The reactions of a variety of aromatic/aliphatic alcohol, malononitrile and dimedone were studied using optimum reaction conditions (Fig. 10, Table 4). Benzyl alcohol having electron-withdrawing or -releasing groups underwent the reaction with malononitrile and dimedone with high efficiency to give 2-amino-3-cyano-4*H*-chromenes in good to high yields. Based on these results, the reaction progress was not sensitive to the electron density of the substrates. Several substituents on the benzyl alcohol such as methoxy, methyl, nitro, chloride and bromide were remained intact during the reaction (Table 4, entries 1–10). The reaction of furfuryl alcohol as a heteroaromatic alcohol and cinnamyl alcohol as an α,β-unsaturated alcohol progressed well (Table 4, entries 11 and 12). 1-Octanol as an aliphatic alcohol was also condensed with malononitrile and dimedone successfully (Table 4, entry 13). Moreover, the reaction of β-dicarbonyl compounds such as cyclohexane-1,3-dione, pentane-2,4-dione, methyl acetoacetate and ethyl acetoacetate were investigated using the present method and desired products were obtained in good yields (Table 4, entries 14–17). The reactions are clean without formation of any side products especially the overoxidation products such as carboxylic acids or esters, which can be formed from alcohols during oxidation reaction. These observations showed the high catalytic activity and selectivity of the catalyst.

**Table 4:** Synthesis of chrome-derivatives via multicomponent TOP catalyzed by (γ-Fe_2_O_3_-Im-Py)_2_WO_4_.

Entry	R	β-dicarbonyl compound	Time^a^ (I + II, h)	Isolated yields (%)	M.P. (°C)	
					Obtained	Reported^ref^
1	C_6_H_5_	Dimedone	1.5 + 4	91	224–226	226–227^76^
2	4-CH_3_-C_6_H_4_	Dimedone	2 + 7	84	225–227	223–225^77^
3	4-OCH_3_-C_6_H_4_	Dimedone	2 + 7	85	191–193	190–192^76^
4	2-Cl-C_6_H_4_	Dimedone	1.5 + 6	89	214–216	214–215^78^
5	4-Cl-C_6_H_4_	Dimedone	1.5 + 5	90	238–240	237–239^78^
6	2,6-Cl_2_-C_6_H_4_	Dimedone	2.5 + 6	86	235–238	236–238^79^
7	4-Br-C_6_H_4_	Dimedone	2 + 7	83	202–204	203–205^77^
8	2-NO_2_-C_6_H_4_	Dimedone	3 + 6	84	227–229	228–229^78^
9	3-NO_2_-C_6_H_4_	Dimedone	3 + 7	83	210–211	212–214^76^
10	4-NO_2_-C_6_H_4_	Dimedone	3 + 6	86	178–179	179–180^77^
11	2-furyl	Dimedone	2.5 + 5.5	88	227–229	226–228^77^
12	C_6_H_5_CH = CH	Dimedone	3 + 5	82	183–186	182–184^78^
13^b^	CH_3_(CH_2_)_6_	Dimedone	10 + 9	45	185–186	187–189^70^
14	C_6_H_5_	Cyclohexane-1,3-dione	1.5 + 5	89	238–240	239–241^77^
15	C_6_H_5_	Pentane-2,4-dione	1.5 + 5	85	161–163	162–163^70^
16	C_6_H_5_	Methyl acetoacetate	1.5 + 7	84	194–196	192–194^70^
17	C_6_H_5_	Ethyl acetoacetate	1.5 + 6	87	190–191	192–194^78^

^a^Reaction conditions: (I) benzyl alcohol (1 mmol), TBHP (6 mmol), (γ-Fe_2_O_3_-Im-Py)_2_WO_4_ (142 mg, 5 mol%; based on W content and relative to alcohol), and (II) malononitrile (1.2 mmol) and β-dicarbonyl compounds (1.2 mmol). ^b^(γ-Fe_2_O_3_-Im-Py)_2_WO_4_ (171 mg, 6 mol%; based on W content and relative to alcohol).

**Figure 11:**
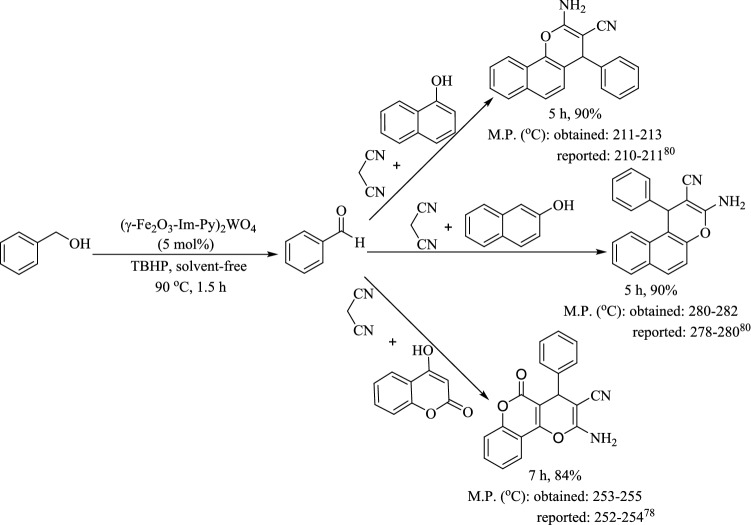
Multicomponent TOP of benzyl alcohol, malononitrile and α-naphthol/β-naphthol/4-hydrxycoumarin catalyzed by (γ-Fe_2_O_3_-Im-Py)_2_WO_4_.

In addition, the applicability of this protocol was evaluated for the activated compounds such as α-naphthol, β-naphthol and 4-hydrxycoumarin (Fig. 11) and the products were obtained in good to high yields.

**Figure 12:**
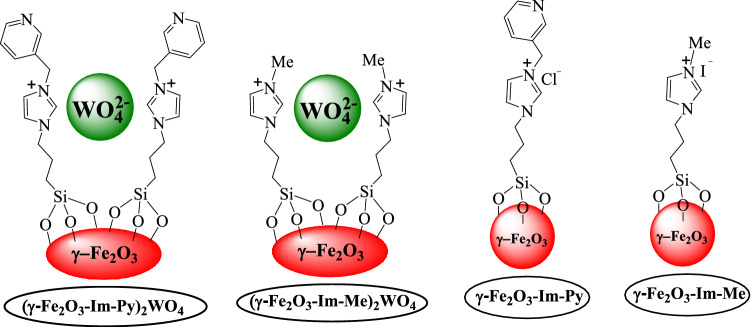
Chemical structure of (γ-Fe_2_O_3_-Im-Py)_2_WO_4_, (γ-Fe_2_O_3_-Im-Me)_2_WO_4_, γ-Fe_2_O_3_-Im-Py and γ-Fe_2_O_3_-Im-Me.

**Figure 13:**
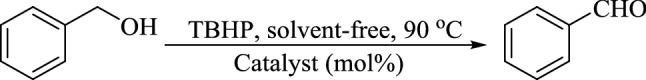
Role of the catalyst in the oxidation of benzyl alcohol.

To prove the role of the catalyst during the oxidation reaction, the oxidation reaction of benzyl alcohol was assessed in the presence of (γ-Fe_2_O_3_-Im-Me)_2_WO_4_ (pyridine-free catalyst), γ-Fe_2_O_3_-Im-Py (tungstate-free catalyst) and γ-Fe_2_O_3_ (Figs. 12 and 13). It was found that the reactions were proceeded in 98, 27 and 27% yields, respectively (Table 5, entries 1–3). These results showed the special effect of tungstate in the oxidation reaction and any role of pyridine in this process. A similar reaction in the presence of sodium tungstate produced the desired product in low yield (24 h, 41%) (Table 5, entry 4), which showed the activation effect of supported imidazolium species on the tungstate groups. Moreover, the reaction under catalyst-free conditions or in the presence of pyridine produced only a trace amount of the product in 24 h (Table 5, entries 5 and 6). The oxidation reaction of benzyl alcohol in presence of (γ-Fe_2_O_3_-Im-Py)_2_MoO_4_ and γ-Fe_2_O_3_-Im-Py-VO_3_, varying two different oxidizing anions, was also examined (Table 5, entries 7 and 8) and the same results as in the presence of (γ-Fe_2_O_3_-Im-Py)_2_WO_4_ were obtained.

**Table 5:** The effect of different catalysts on the oxidation of benzyl alcohol.

Entry	Catalyst	Time (h)	Yield^a^ (%)
1^b^	(γ-Fe_2_O_3_-Im-Me)_2_WO_4_ (2 mol%, 57 mg)	3	98
2	γ-Fe_2_O_3_-Im-Py (57 mg)	24	27
3	γ-Fe_2_O_3_ (57 mg)	24	27
4^b^	Na_2_WO_4_.2H_2_O (2 mol%, 6.6 mg)	24	41
5	Py (2 mol%, 1.58 mg)	24	Trace
6	–	24	Trace
7^b^	(γ-Fe_2_O_3_-Im-Py)_2_MoO_4_ (2 mol%, 64 mg)	3	96
8^b^	γ-Fe_2_O_3_-Im-Py-VO_3_ (2 mol%, 31 mg)	3	98

^a^Isolated yield. Reaction conditions: benzyl alcohol (1 mmol), TBHP (6 mmol) at 90 °C. ^b^Amount of the catalyst is based on W, Mo or V contents, relative to benzyl alcohol.

**Figure 14:**
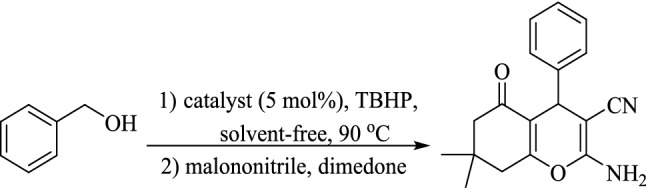
Role of the catalyst in the synthesis of 2-amino-3-cyano-4*H*-chromene from benzyl alcohol.

To demonstrate the role of the catalyst in the TOP synthesis of 2-amino-3-cyano-4*H*-chromene (Fig. 14), the model reaction was surveyed with (γ-Fe_2_O_3_-Im-Me)_2_WO_4_ as the pyridine-free analogues catalyst (Fig. 12). The product was obtained in a moderate yield (50%), which shows the importance of the pyridine effect on the Knoevenagel condensation-Michael addition-cyclization reaction of the *in-situ* formed aldehyde with malononitrile and dimedone (Table 6, entry 2).

**Table 6:** The effect of different catalysts in the synthesis of 2-amino-3-cyano-4*H*-chromene from benzyl alcohol.

Entry	Catalyst^a^ (mol%)	Time (I + II, h)	Yield^b^(%)
1	(γ-Fe_2_O_3_-Im-Py)_2_WO_4_	1.5 + 4	91
2	(γ-Fe_2_O_3_-Im-Me)_2_WO_4_	1.5 + 6	50

^a^Amount of the catalyst (5 mol%, 142 mg) is based on W content and relative to benzyl alcohol. ^b^Reaction conditions: (I) benzyl alcohol (1 mmol), TBHP (6 mmol), catalyst, neat, 90 °C; and (II) malononitrile (1.2 mmol), dimedone (1.2 mmol), neat, 90 °C.

**Figure 15:**
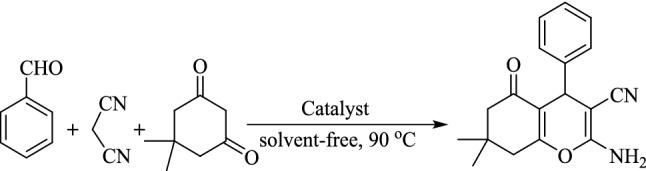
Role of the catalyst in the synthesis of 2-amino-3-cyano-4*H*-chromene from benzaldehyde.

The effect of the catalyst in the Knoevenagel condensation-Michael addition-cyclization step of the synthesis of 2-amino-3-cyano-4*H*-chromenes was also studied by performing the reaction of benzaldehyde, malononitrile and dimedone (Fig. 15) using (γ-Fe_2_O_3_-Im-Py)_2_WO_4_, γ-Fe_2_O_3_-Im-Py (tungstate-free catalyst) and γ-Fe_2_O_3_-Im-Me (tungstate and pyridine-free catalyst) (Fig. 12, Table 7). Any effect of tungstate was not observed in the Knoevenagel condensation-Michael addition-cyclization reaction. Therefore, in this step, the most active site of the catalyst should be pyridine and imidazolium moiety.

**Table 7:** The effect of different catalysts in the synthesis of 2-amino-3-cyano-4*H*-chromene from benzaldehyde.

Entry	Catalyst	Time (h)	Yield^a^ (%)
1^b^	(γ-Fe_2_O_3_-Im-Py)_2_WO_4_	1.5	95
2	γ-Fe_2_O_3_-Im-Py	1.5	95
3	γ-Fe_2_O_3_-Im-Me	2.5	50

^a^Reaction conditions: benzaldehyde (1 mmol), malononitrile (1.2 mmol), dimedone (1.2 mmol), catalyst (142 mg), neat, 90 °C. ^b^Amount of the catalyst (5 mol%, 142 mg) is based on W content and relative to alcohol.

**Figure 16:**
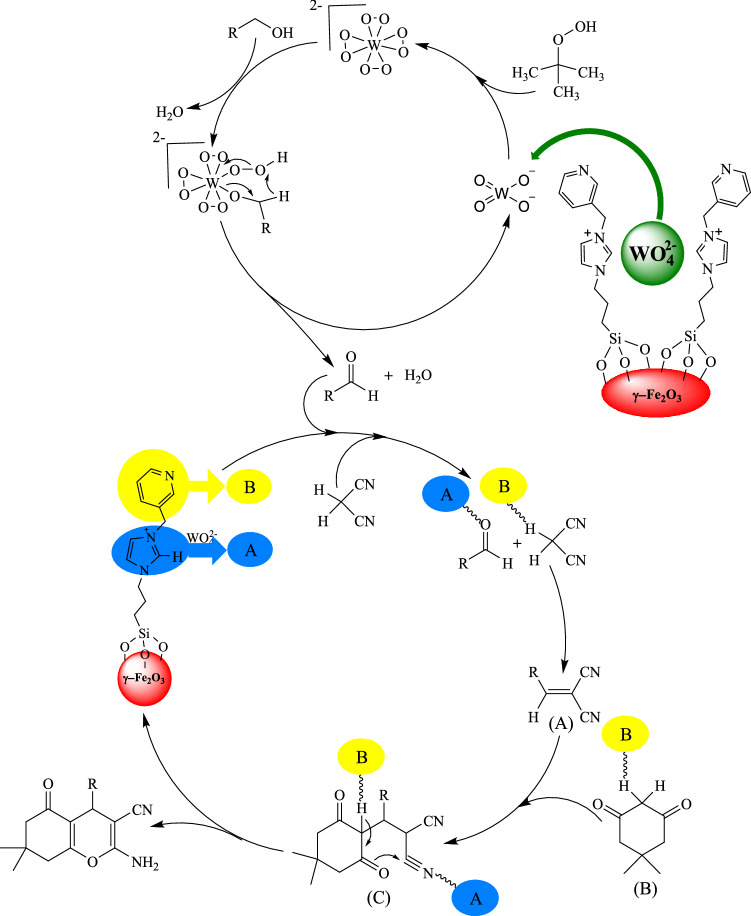
Proposed mechanism pathway for the preparation of functionalized-4*H*-chromenes via a multicomponent TOP catalyzed by (γ-Fe_2_O_3_-Im-Py)_2_WO_4_ as a multifunctional catalyst.

A plausible mechanism was estimated for the reaction based on our results and proposed mechanisms in the literature^81–84^. Firstly, aldehydes are produced from alcohols by the dehydration in the presence of TBHP catalysed by tungstate ions. In the next step, the catalyst enables the formation of dicyanoolefins (A) by Knoevenagel condensation of in-situ formed aldehydes with malononitrile. Then, Michael addition of enolate of dimedone (B) to dicyanoolefins leads to the formation of C, followed by cyclocondensation and tautomerization to form 2-amino-3-cyano-4*H*-chromenes (Fig. 16). During this process, imidazolium cations activate electrophiles (aldehyde and malononitrile) by hydrogen-bond formation between the carbonyl and nitrile groups with the hydrogen at the 2-position of the imidazolium ring. At the same time, pyridine activates nucleophiles by removing the acidic hydrogens from these compounds. The dual activation of nucleophiles and electrophiles by the imidazolium and pyridine is essential to promote the reaction in good to high yields. This activation effect can be clearly observed in the synthesis of 2-amino-3-cyano-4*H*-chromenes from the *in-situ* formed aldehydes containing electron-resealing or electron-withdrawing groups in good to high yields, regardless of the electron density of the substrates (Table 4, entries 1–10).

**Figure 17:**
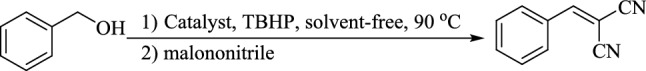
Reaction between benzyl alcohol and malononitrile for the synthesis of 2-benzylidenemalononitrile.

**Figure 18:**
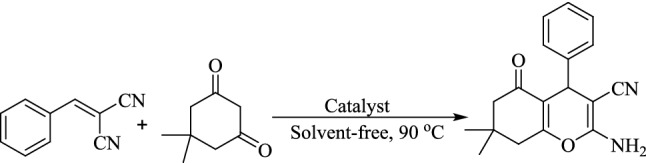
Reaction between 2-benzylidenemalononitrile and dimedone for synthesis of 2-amino-3-cyano-4*H*-chromene.

The probability of formation of dicyanoolefins (A) (Fig. 16) as an intermediate in the reaction was studied by performing the catalytic reaction of benzyl alcohol with malononitrile in the presence of (γ-Fe_2_O_3_-Im-Py)_2_WO_4_ under optimized reaction conditions (Fig. 17). 2-Benzylidenemalononitrile, was isolated in 93% yield (Table 8, entry 1) and characterized by its ^1^H NMR spectrum (Supplementary Fig. S15). A reaction between 2-benzylidenemalononitrile and dimedone in the presence of catalyst was also conducted (Fig. 18) and 2-amino-3-cyano-4*H*-chromene was isolated in 96% yield (Table 9, entry 1). These results clearly confirmed the formation of Knoevenagel condensation product (A), as an intermediate in the TOP synthesis of 2-amino-3-cyano-4*H*-chromenes from alcohols. Similar reactions in the presence of (γ-Fe_2_O_3_-Im-Me)_2_WO_4_ as pyridine-free analogue of the catalyst produced the desired product in lower yield (Tables 8 and 9, entry 2), which showed the special role of pyridine as a base in the Knoevenagel and sequential Michael addition-cyclization reactions.

**Table 8:** The effect of different catalysts in the synthesis of 2-benzylidenemalononitrile from benzyl alcohol.

Entry	Catalyst^a^	Time (I + II, h)	Yield^b^ (%)
1	(γ-Fe_2_O_3_-Im-Py)_2_WO_4_	1.5 + 2.5	93
2	(γ-Fe_2_O_3_-Im-Me)_2_WO_4_	1.5 + 5.5	57

^a^Amount of the catalyst is based on W content and relative to benzyl alcohol. ^b^Reaction conditions: (I) benzyl alcohol (1 mmol), TBHP (6 mmol), catalyst (5 mol%, 142 mg), neat, 90 °C; and (II) malononitrile (1.2 mmol).

**Table 9:** The effect of different catalysts in the synthesis of 2-amino-3-cyano-4*H*-chromene from benzyl alcohol.

Entry	Catalyst^a^ (mol%)	Time (min)	Yield^b^ (%)
1	(γ-Fe_2_O_3_-Im-Py)_2_WO_4_	30	96
2	(γ-Fe_2_O_3_-Im-Me)_2_WO_4_	90	73

^a^Amount of the catalyst is based on W content and relative to benzyl alcohol. ^b^Reaction conditions: 2-benzylidenemalononitrile (1 mmol), dimedone (1 mmol), catalyst (5 mol%, 142 mg), neat, 90 °C.

**Figure 19:**
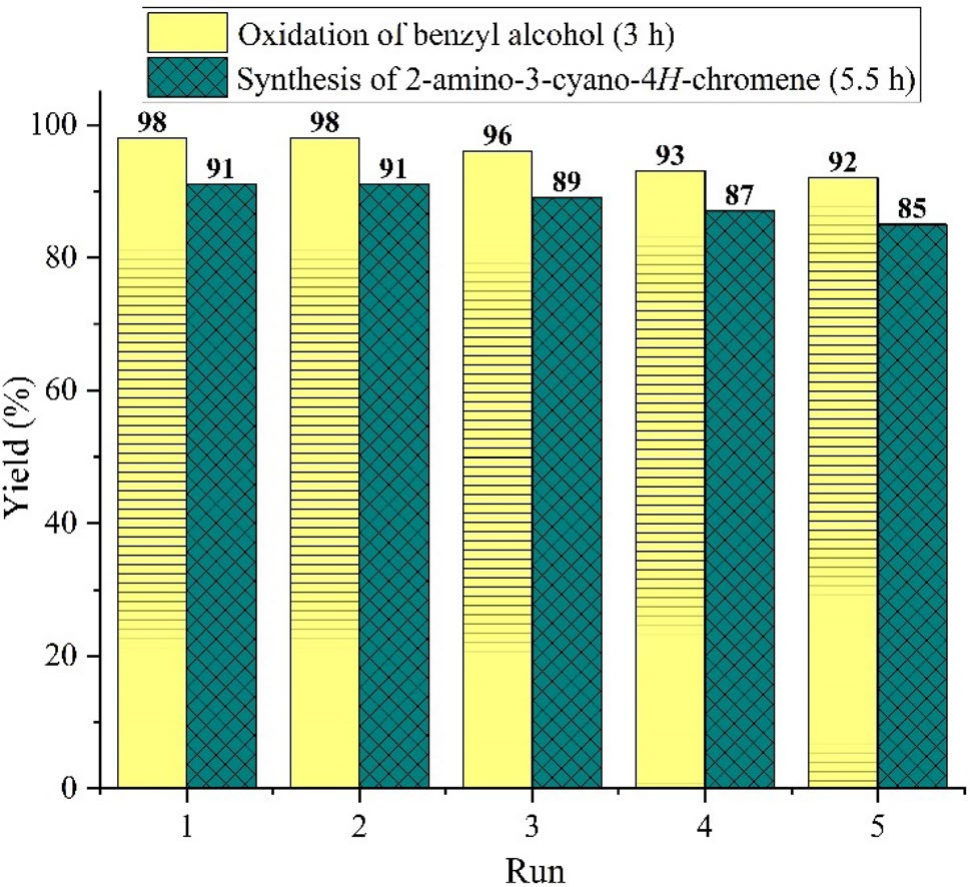
Recyclability of (γ-Fe_2_O_3_-Im-Py)_2_WO_4_ catalyst in the oxidation of benzyl alcohol and also in the preparation of functionalized 2-amino-3-cyano-4*H*-chromene via multicomponent TOP in the model reaction, under optimized reaction conditions.

**Figure 20:**
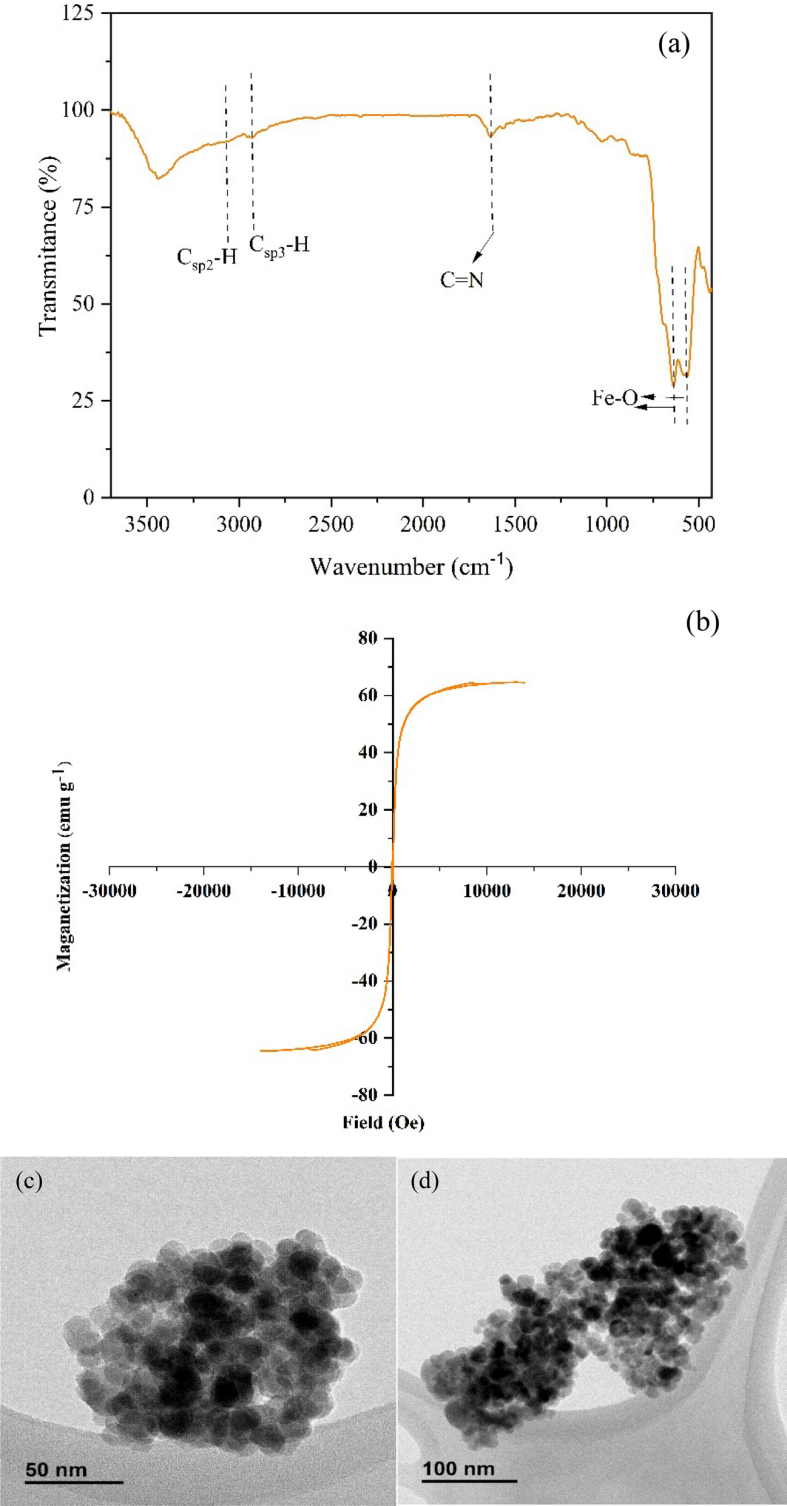
(**a**) FT-IR spectrum (**b**) VSM curve and (c,d) TEM images of (γ-Fe_2_O_3_-Im-Py)_2_WO_4_ after 5th run reuse in the synthesis of 2-amino-3-cyano-4*H*-chromenes via multicomponent TOP.

The catalyst recyclability and reusability were examined in the oxidation of benzyl alcohol and also in the preparation of functionalized 2-amino-3-cyano-4*H*-chromene via multicomponent TOP in the model reaction, under optimized reaction conditions. Ethyl acetate was added to the completed reaction and (γ-Fe_2_O_3_-Im-Py)_2_WO_4_ was isolated simply using an external magnet, washed with EtOAc and EtOH. Then, the catalyst dried in a vacuum oven, and recycled again for another new batch. After five consecutive runs, the catalyst still showed high catalytic performance (Fig. 19). To demonstrate that (γ-Fe_2_O_3_-Im-Py)_2_WO_4_ is truly heterogeneous, a leaching experiment was directed. Analysis of the reaction mixture by ICP after catalyst separation showed that the leaching was very low, so that after the 5^th^ recovery, the leaching amount of tungsten was less than 0.3 ppm. The result of ICP showed that (γ-Fe_2_O_3_-Im-Py)_2_WO_4_ is truly heterogeneous and catalyst leaching is negligible under this reaction condition. FT-IR (Fig. 20a), VSM (Fig. 20b) and TEM images (Fig. 20c,d) of the recycled (γ-Fe_2_O_3_-Im-Py)_2_WO_4_ after five runs also revealed the significant stability of the catalyst.

Considering the importance of large-scale reactions, in the last part, we have evaluated the scalability of the oxidation reaction and muticomponent TOP synthesis of 2-amino-3-cyano-4*H*-chromenes. To do this, the oxidaton reaction of benzyl alcohol and also muticomponent TOP of benzyl alcohol, malononitrile and dimedone in a scaled-up procedure (50 times) in the presence of (γ-Fe_2_O_3_-Im-Py)_2_WO_4_ was carried out successfully under the optimized reaction conditions. Interestingly, the scaled-up reaction accompanied with 95 and 88% isolated yields of the desired products.

## Conclusion

In this study, we have designed and synthesized a novel base-metal multifunctional catalyst [(γ-Fe_2_O_3_-Im-Py)_2_WO_4_]. This multifunctional heterogeneous nanocatalyst is completely characterized by various techniques such as FT-IR, XPS, TEM, FESEM, ICP, TGA, VSM and XRD. Then, its catalytic activity was evaluated in the synthesis of 2-amino-3-cyano-4*H*-chromenes via a multicomponent tandem oxidation process starting from alcohols as suitable alternatives for integrate consideration of economic viability and environmental integrity. Different types of 2-amino-3-cyano-4*H*-chromenes were produced in good to high yields by the reaction of different types of in-situ formed aldehydes with malononitrile, and β-dicarbonyl compounds/naphthols/4-hydroxycumarin under solvent-free conditions. The catalyst operated by a dual activation of nucleophiles and electrophiles and was readily separated from the reaction mixture and reused in five cycles with high degree of efficiency. The high efficiency of the catalyst is related to the tandem catalytic effect of tungstate in the oxidation of alcohols and the basic role of pyridine and imidazolium sites in the Knoevenagel condensation-Michael addition-cyclization reaction of in-situ formed aldehydes with malononitrile and activated nucleophilic components.

## Supplementary Information

Supplementary Figures.
