# Supplementary material for: A novel base-metal multifunctional catalyst for the synthesis of 2-amino-3-cyano-4H-chromenes by a multicomponent tandem oxidation process
Source: Sci Rep. 2022 Feb 21;12:2867. doi: 10.1038/s41598-022-06759-7 (PMC8861043; doi:10.1038/s41598-022-06759-7)
Supplement: Supplementary file 1 — Supplementary Figures. [file 41598_2022_6759_MOESM1_ESM.pdf]

Supplementary Information

A Novel Base-Metal Multifunctional Catalyst for the Synthesis of 2-Amino-3-Cyano-4*H*-Chromenes by a Multicomponent Tandem Oxidation Process

Farhad Omarzehi Chahkamali,[a] Sara Sobhani*[a] and Jose Miguel Sansano[b]

[a]Department of Chemistry, College of Sciences, University of Birjand, Birjand, Iran, e-mail: ssobhani@birjand.ac.ir, sobhanisara@yahoo.com, phone: +985632016660.

[b]Departamento de Química Orgánica, Facultad de Ciencias, Centro de Innovación en Química Avanzada (ORFEO-CINQA), Universidad de Alicante, Apdo. 99, 03080-Alicante, Spain.

General information

NMR spectra were recorded in ppm in $CDCl_3$ and DMSO-d_6 using a Bruker Advance 300 MHz instrument with TMS as the internal standard.

Spectral Data:

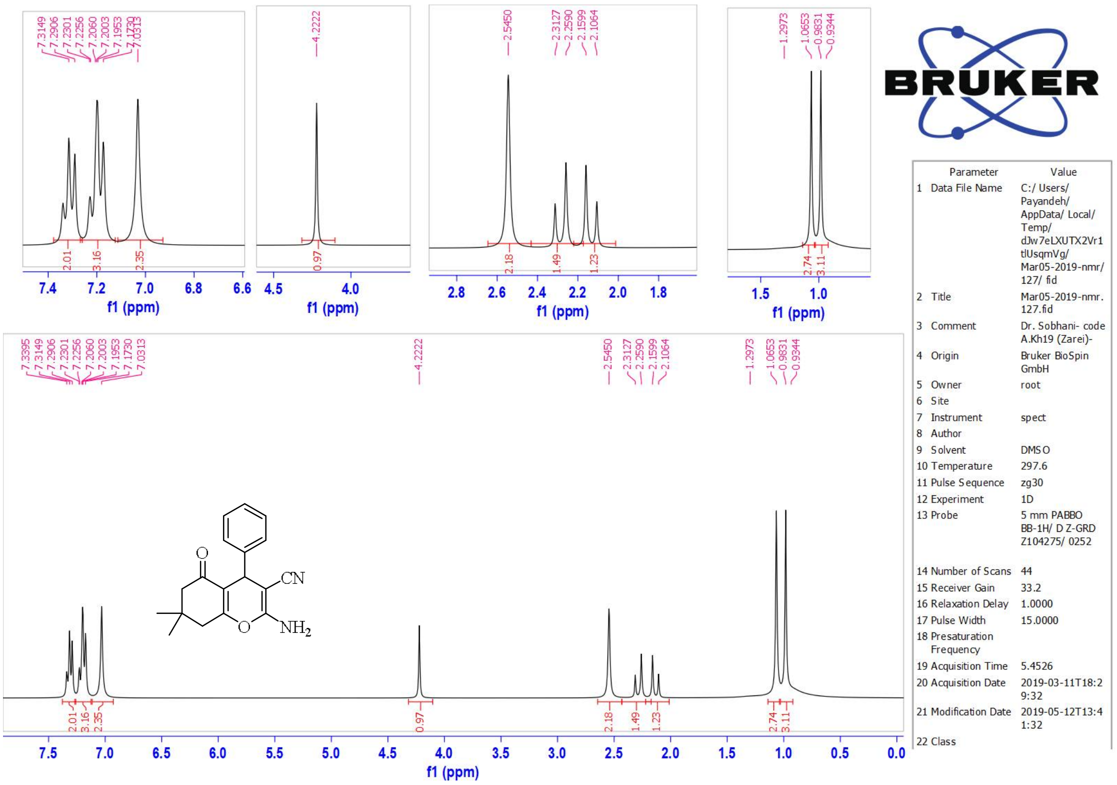

Figure S1. ^{1}H NMR spectrum of 2-amino-4-(phenyl)-7,7-dimethyl-5-oxo-5,6,7,8-tetrahydro-4*H*-chromene-3-carbonitrile (Table 4, entry 1)

^{1}H NMR (300 MHz, DMSO-d$_6$): δ 7.29-7.33 (m, 2 H), 7.17-7.23 (m, 3 H), 7.03 (s, 2 H), 4.22 (s, 1 H), 2.54 (s, 2 H), 2.28 (d, *J* =16.1 Hz, 1 H), 2.13 (d, *J* = 16.0 Hz, 1 H), 1.06 (s, 3 H), 0.98 (s, 3 H) ppm.

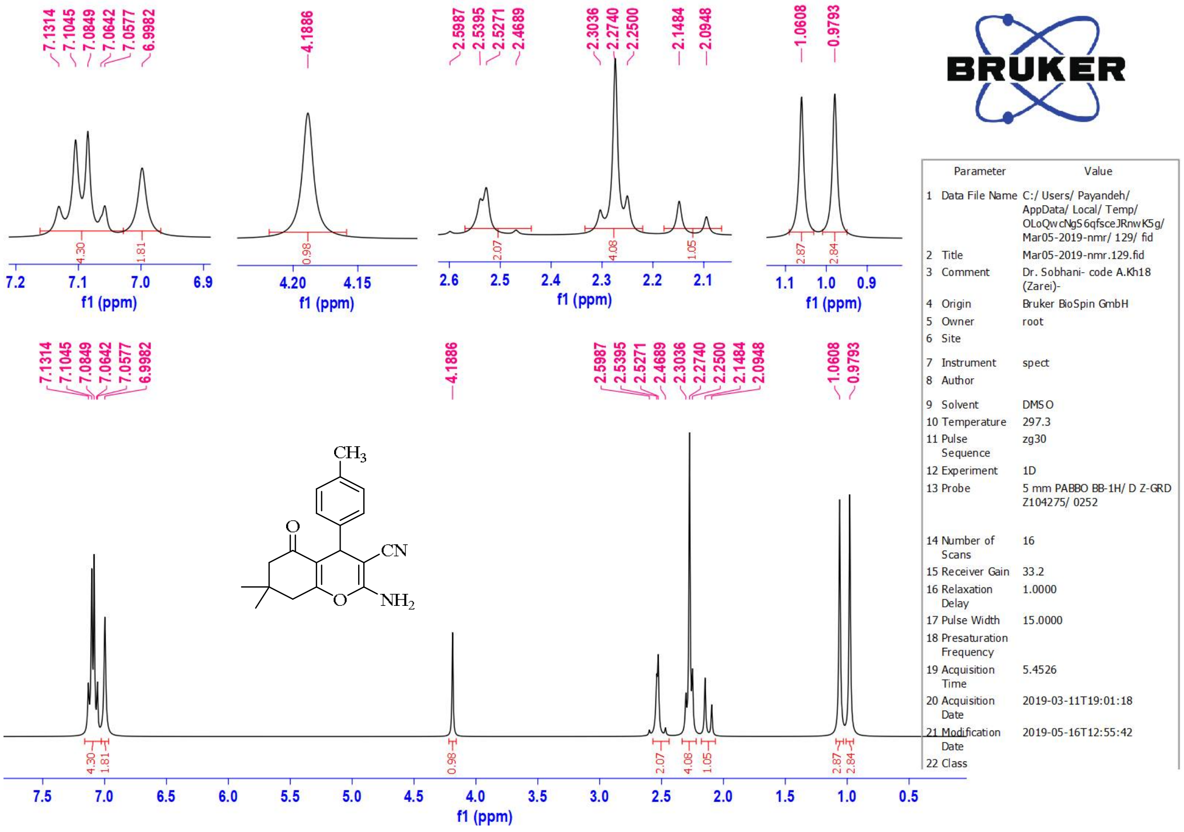

Figure S2. ^{1}H NMR spectrum of 2-amino-4-(p-tolyl)-7,7-dimethyl-5-oxo-5,6,7,8-tetrahydro-4*H*-chromene-3-carbonitrile (Table 4, entry 2)

^{1}H NMR (300 MHz, DMSO-d$_6$): δ 7.05-7.13 (m, 4 H), 6.99 (s, 2 H), 4.18 (s, 1 H), 2.57 (d, *J* = 16.1 Hz, 1 H), 2.50 (d, *J* = 17.4 Hz, 1 H), 2.28 (d, *J* = 16.1 Hz, 1 H), 2.27 (s, 3 H), 2.12 (d, *J* = 16.1 Hz, 1 H), 1.06 (s, 3 H), 0.97 (s, 3 H) ppm.

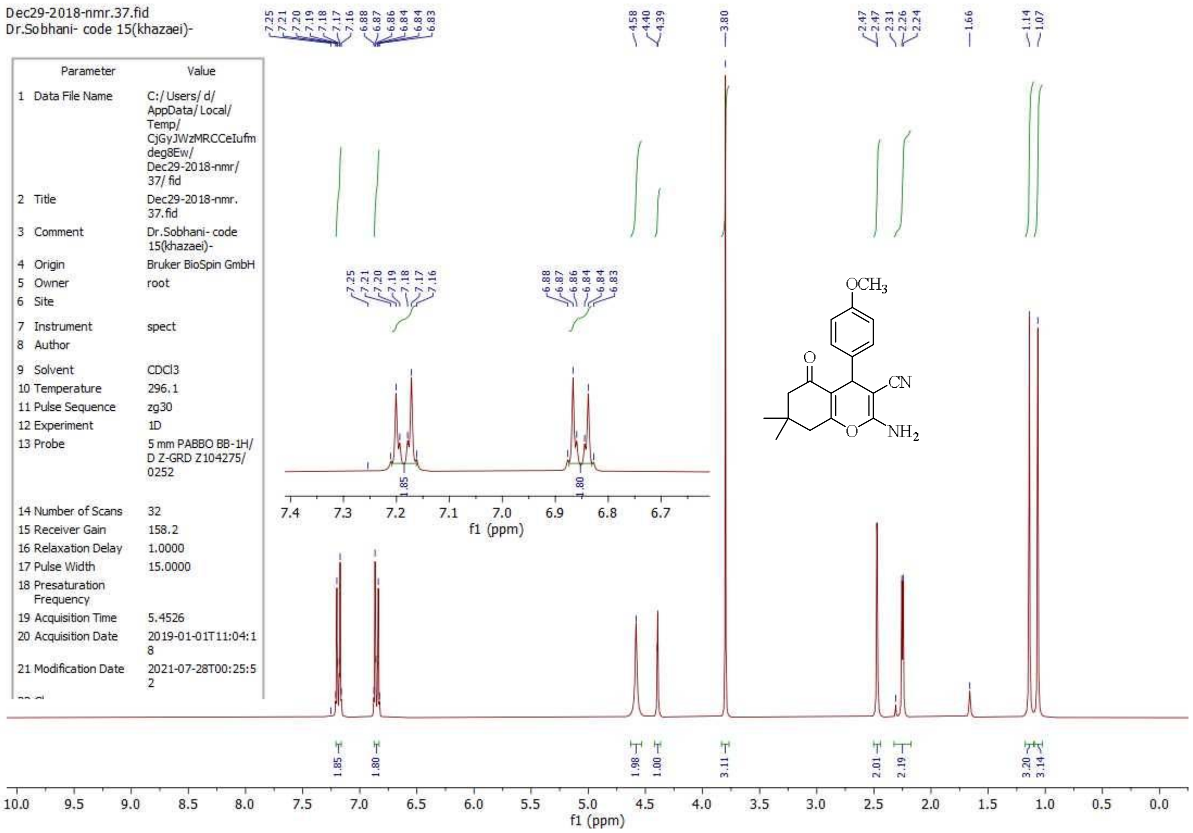

Figure S3. ^{1}H NMR spectrum of 2-amino-4-(4-methoxyphenyl)-7,7-dimethyl-5-oxo-5,6,7,8-tetrahydro-4*H*-chromene-3-carbonitrile (Table 4, entry 3)

^{1}H NMR (300 MHz, $CDCl_3$): δ 7.17-7.20 (m, 2 H), 6.83-6.86 (m, 2 H), 4.58 (s, 2 H), 4.39 (s, 1 H), 3.79 (s, 3 H), 2.47 (s, 2 H), 2.28 (d, J = 16.2 Hz, 1 H), 2.22 (d, J = 16.2 Hz, 1 H), 1.14 (s, 3 H), 1.06 (s, 3 H) ppm.

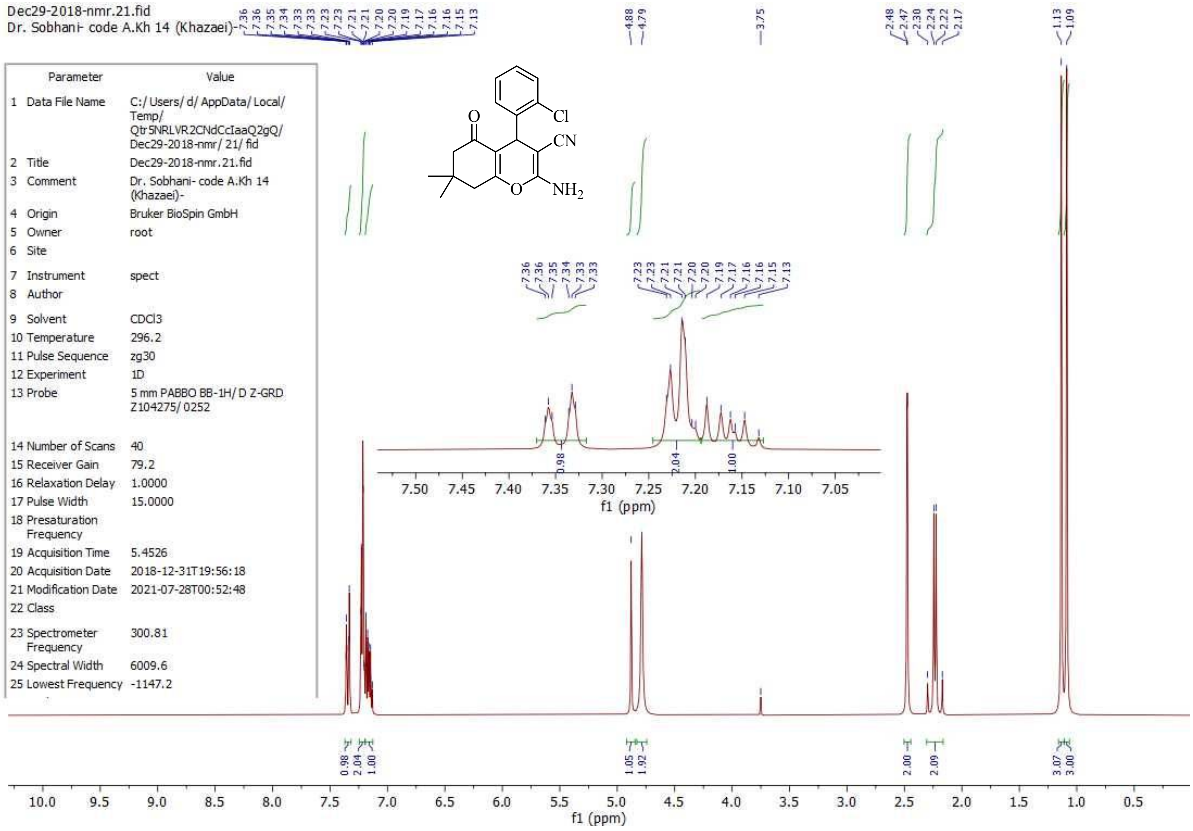

Figure S4. ^{1}H NMR spectrum of 2-amino-4-(2-chlorophenyl)-7,7-dimethyl-5-oxo-5,6,7,8-tetrahydro-4*H*-chromene-3-carbonitrile (Table 4, entry 4)

^{1}H NMR (300 MHz, $CDCl_3$): δ 7.33-7.36 (m, 1 H), 7.20-7.23 (m, 2 H), 7.13-7.18 (m, 1 H), 4.88 (s, 1 H), 4.79 (s, 2 H), 2.48 (s, 2 H), 2.27 (d, J = 16.2 Hz, 1 H), 2.20 (d, J = 16.2 Hz, 1 H), 1.13 (s, 3 H), 1.09 (s, 3 H) ppm.

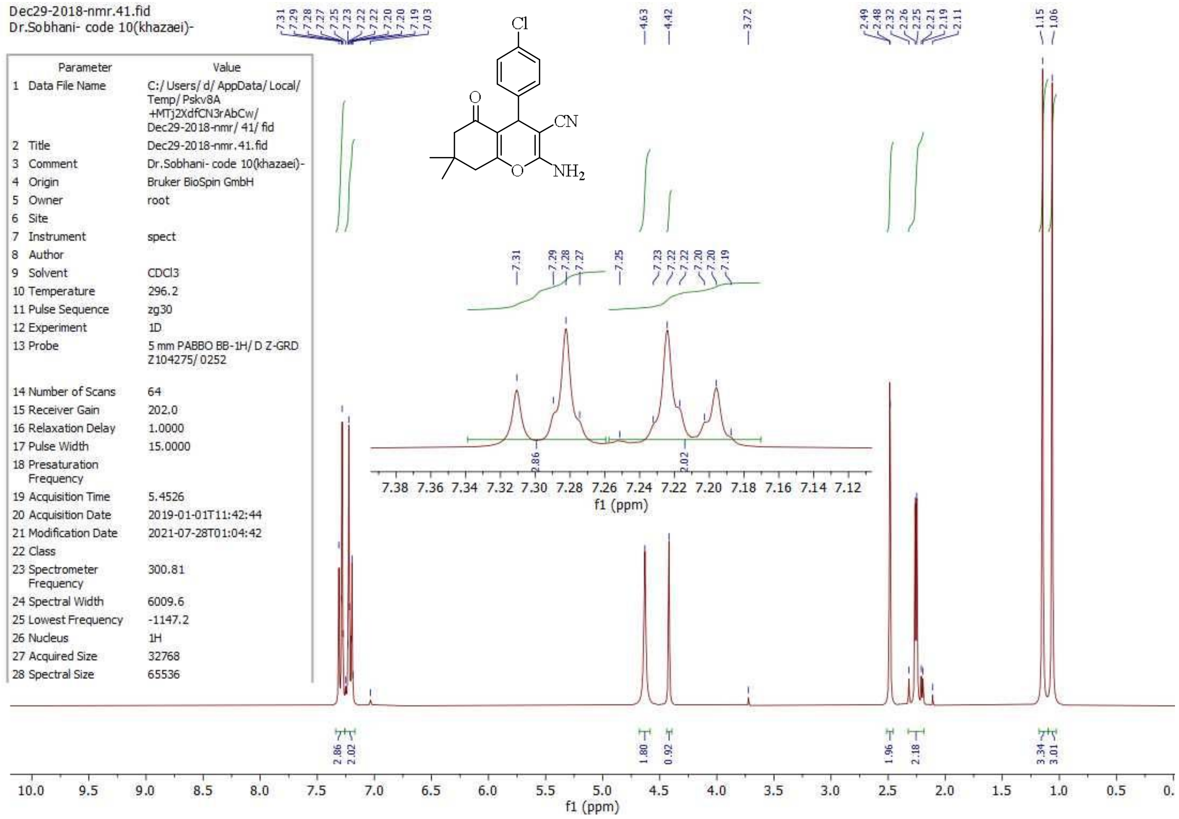

Figure S5. ^{1}H NMR spectrum of 2-amino-4-(4-chlorophenyl)-7,7-dimethyl-5-oxo-5,6,7,8-tetrahydro-4*H*-chromene-3-carbonitrile (Table 4, entry 5)

^{1}H NMR (300 MHz, $CDCl_3$): δ 7.28-7.31 (m, 2 H), 7.19-7.22 (m, 2 H), 4.63 (s, 2 H), 4.42 (s, 1 H), 2.48 (s, 2 H), 2.29 (d, J = 16.2 Hz, 1 H), 2.22 (d, J = 16.2 Hz, 1 H), 1.15 (s, 3 H), 1.06 (s, 3 H) ppm.

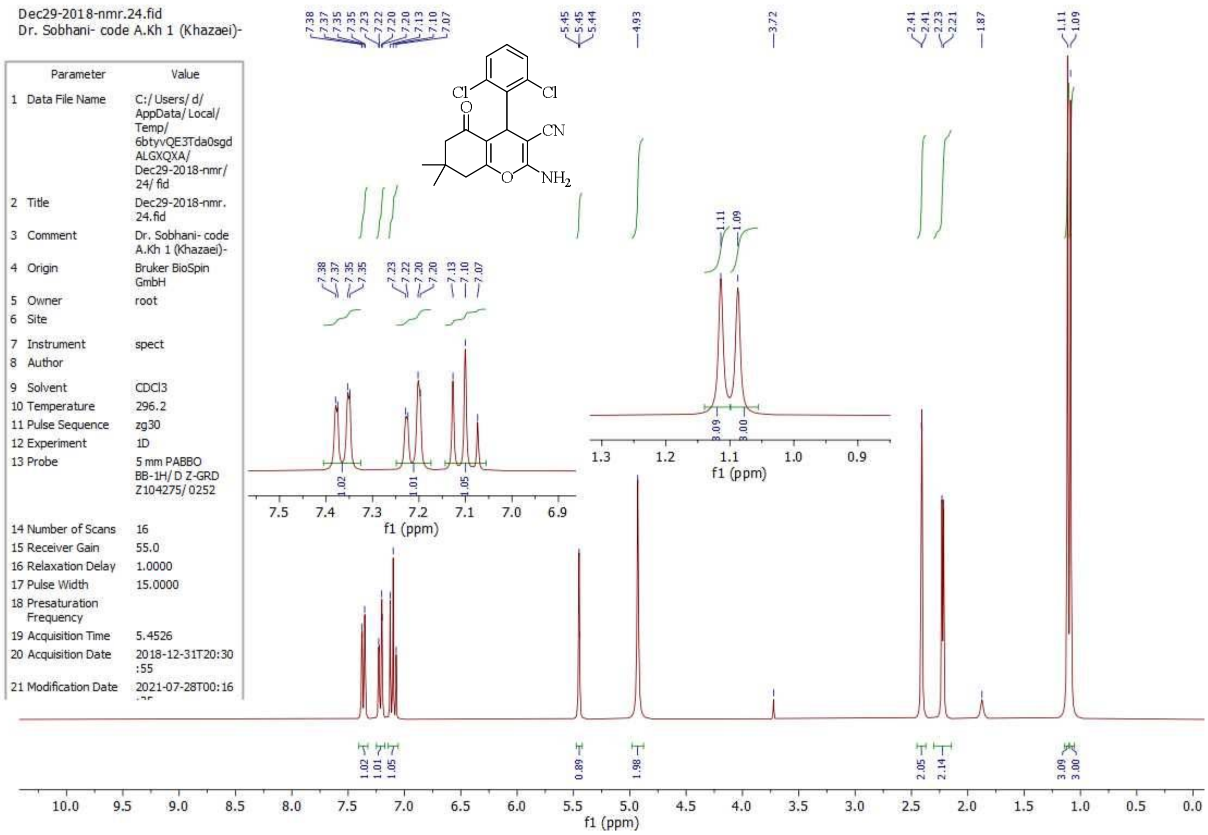

Figure S6. ^{1}H NMR spectrum of 2-amino-4-(2,6-dichlorophenyl)-7,7-dimethyl-5-oxo-5,6,7,8-tetrahydro-4*H*-chromene-3-carbonitrile (Table 4, entry 6)

^{1}H NMR (300 MHz, $CDCl_3$): δ 7.36 (d, J = 7.8 Hz, 1 H), 7.21 (d, J = 7.5 Hz, 1 H), 7.10 (t, J = 7.9 Hz, 1 H), 5.45 (s, 1 H), 4.93 (s, 2 H), 2.41 (s, 2 H), 2.25 (d, J = 16.2 Hz, 1 H), 2.19 (d, J = 16.2 Hz, 1 H), 1.11 (s, 3 H), 1.08 (s, 3 H) ppm.

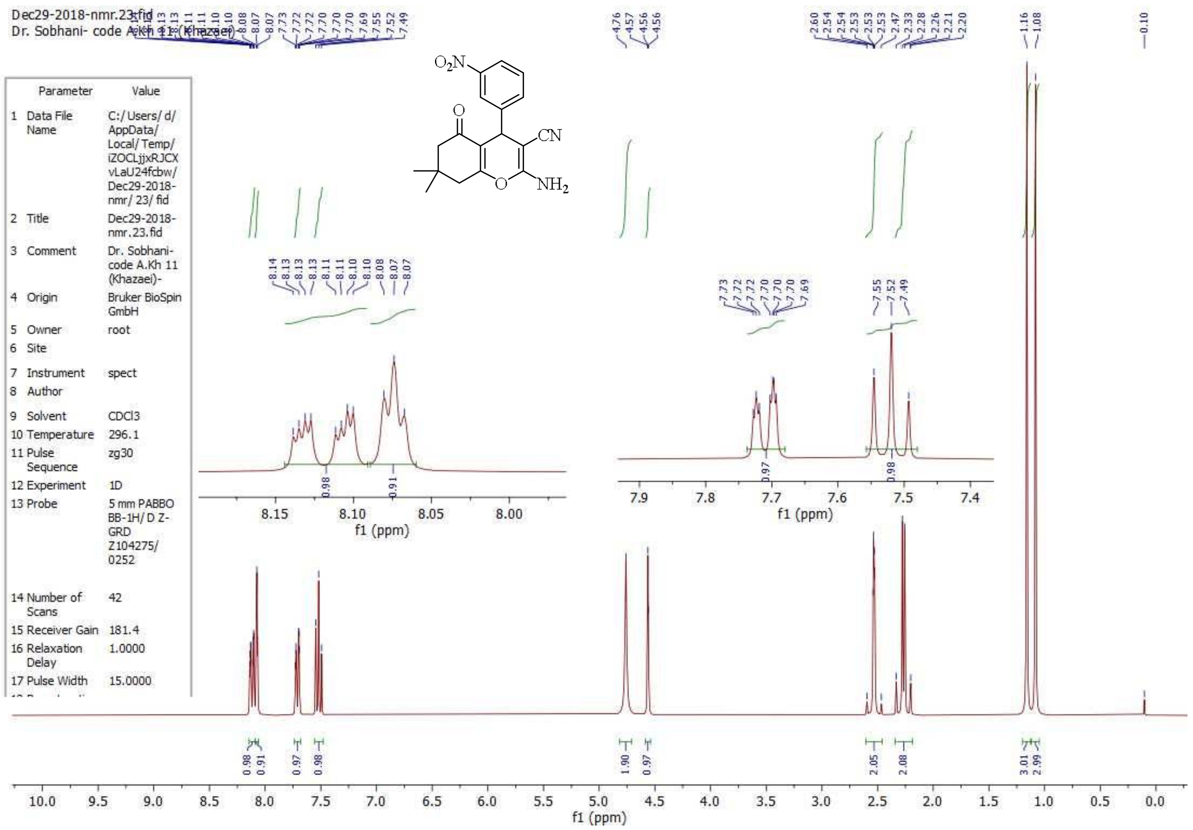

Figure S7. ^{1}H NMR spectrum of 2-amino-4-(3-nitrophenyl)-7,7-dimethyl-5-oxo-5,6,7,8-tetrahydro-4*H*-chromene-3-carbonitrile (Table 4, entry 9)

^{1}H NMR (300 MHz, $CDCl_3$): δ 8.10-8.14 (m, 1 H), 8.06-8.08 (m, 1 H), 7.69-7.73 (m, 1 H), 7.52 (t, *J* = 7.8 Hz, 1 H), 4.76 (s, 2 H), 4.56 (s, 1 H), 2.57 (d, *J* = 17.7 Hz, 1 H), 2.49 (d, *J* = 17.2 Hz, 1 H), 2.30 (d, *J* = 16.3 Hz, 1 H), 2.23 (d, *J* = 16.2 Hz, 1 H), 1.11 (s, 3 H), 1.08 (s, 3 H) ppm.

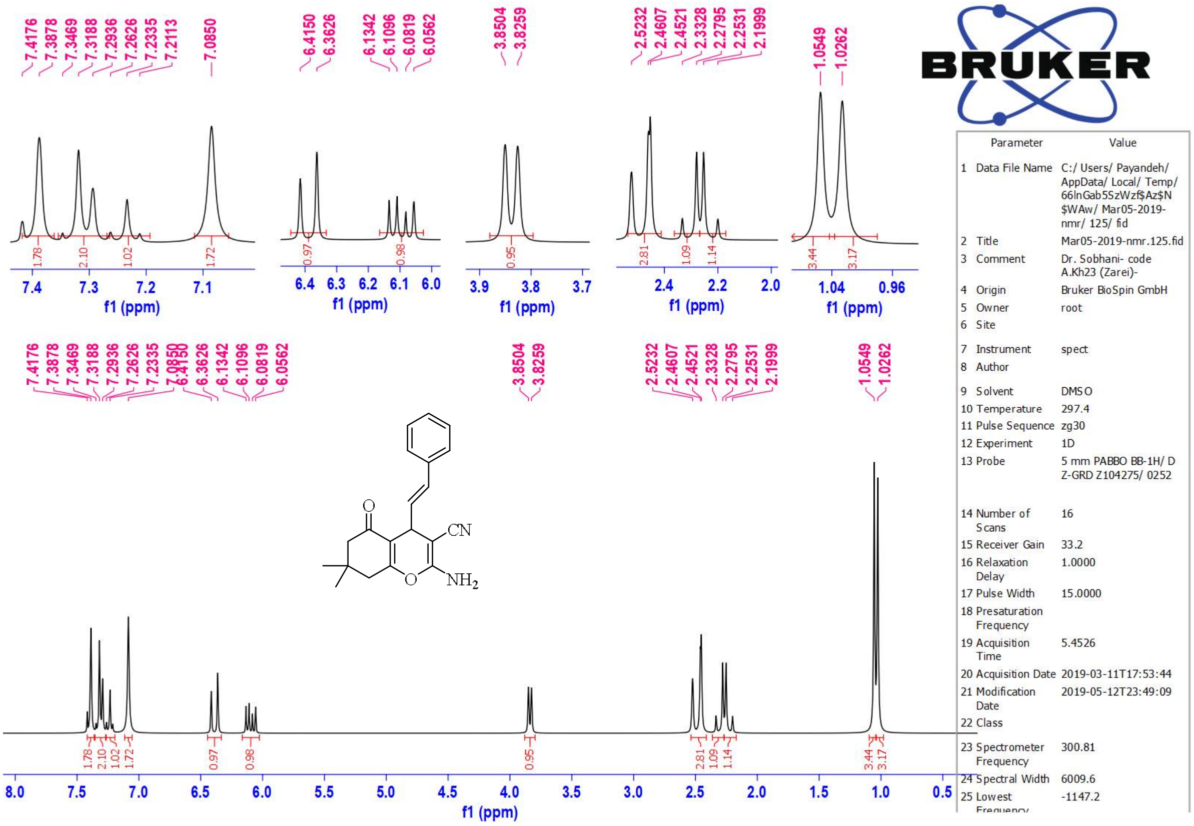

Figure S8. ^{1}H NMR spectrum of 2-amino-7,7-dimethyl-5-oxo-4-styryl-5,6,7,8-tetrahydro-4*H*-chromene-3-carbonitrile (Table 4, entry 12)

^{1}H NMR (300 MHz, DMSO-d$_6$): δ 7.21-7.41 (m, 5 H), 7.08 (s, 2 H), 6.38 (d, *J* = 15.7 Hz, 1 H), 6.09 (dd, *J* = 7.4 Hz, *J* = 15.8 Hz, 1 H), 3.83 (d, *J* = 7.3 Hz, 1 H), 2.45-2.52 (m, 2 H), 2.31 (d, *J* = 16.0 Hz, 1 H), 2.23 (d, *J* = 16.0 Hz, 1 H), 1.05 (s, 3 H), 1.02 (s, 3 H) ppm.

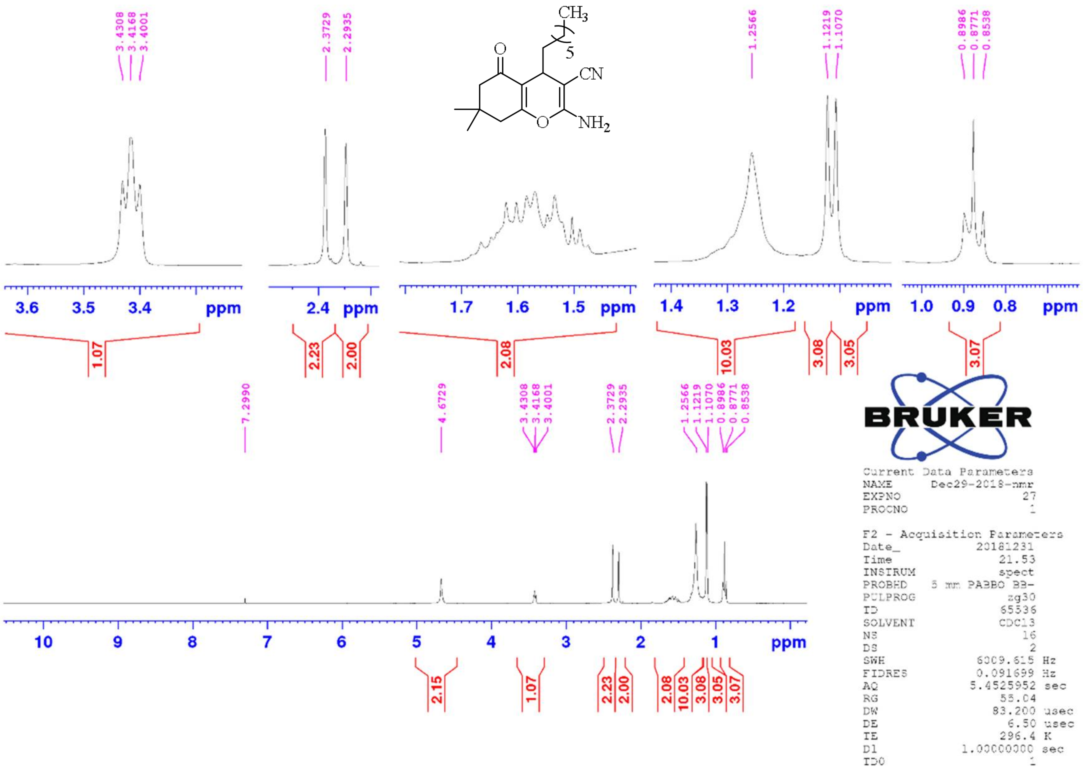

Figure S9. ^{1}H NMR spectrum of 2-amino-4-(heptyl)-7,7-dimethyl-5-oxo-5,6,7,8-tetrahydro-4*H*-chromene-3-carbonitrile (Table 4, entry 13)

^{1}H NMR (300 MHz, $CDCl_3$): δ 4.67 (s, 2 H), 3.40-3.43 (m, 1 H), 2.37 (s, 2 H), 2.29 (s, 2 H), 1.45-1.68 (m, 2 H), 1.25 (bs, 10 H), 1.12 (s, 3 H), 1.10 (s, 3 H), 0.87 (t, J = 6.4 Hz, 3 H) ppm.

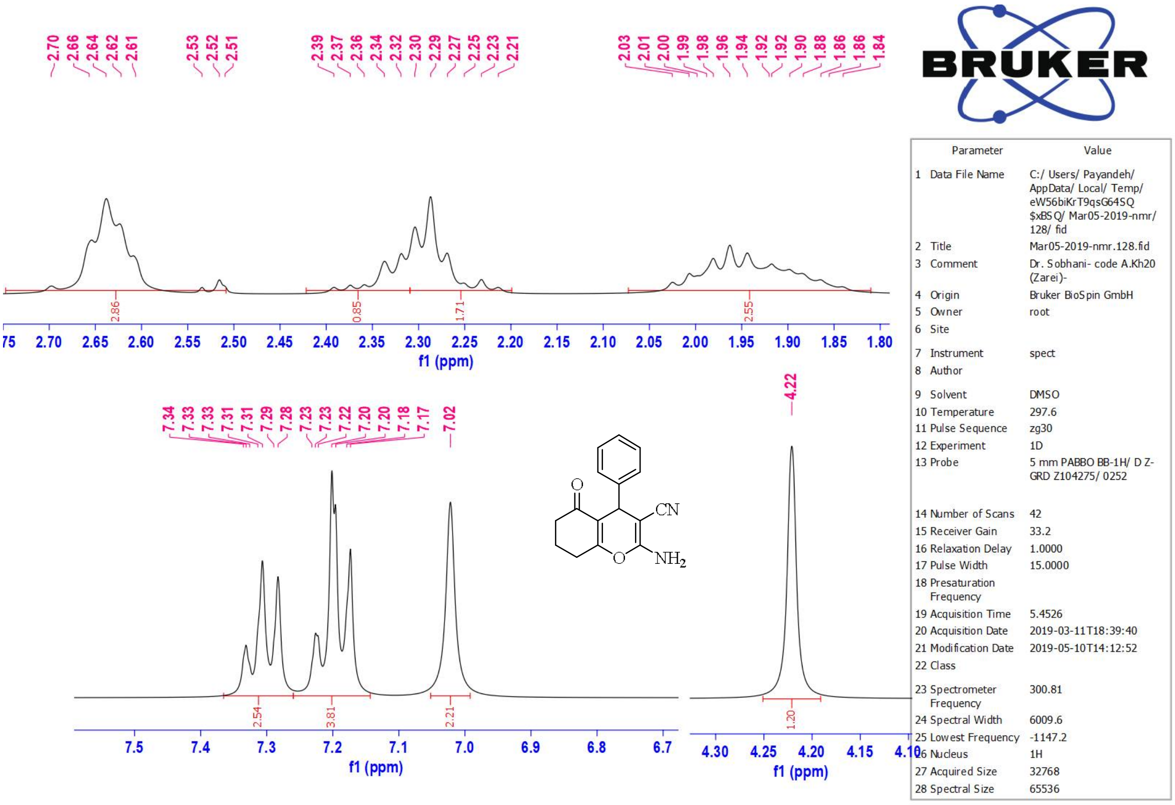

Figure S10. ^{1}H NMR spectrum of 2-amino-5-oxo-4-(phenyl)-5,6,7,8-tetrahydro-4*H*-chromene-3-carbonitrile (Table 4, entry 14)

^{1}H NMR (300 MHz, DMSO-d$_6$): δ 7.28-7.33 (m, 2 H), 7.17-7.23 (m, 3 H), 7.02 (s, 2 H), 4.22 (s, 1 H), 2.50-2.69 (m, 2 H), 2.21-2.39 (m, 2 H), 1.84-2.02 (m, 2 H) ppm.

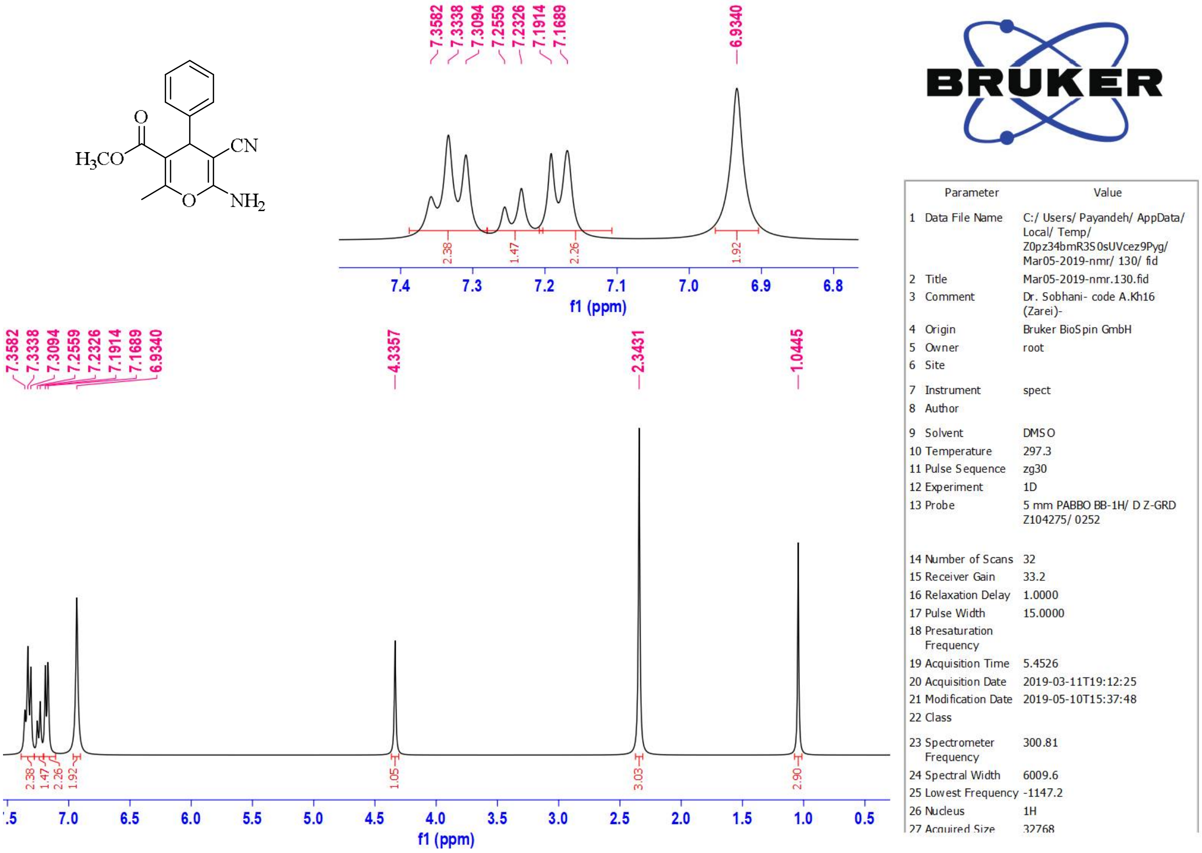

Figure S11. ^{1}H NMR spectrum of methyl 6-amino-5-cyano-2-methyl-4-phenyl-4*H*-chromene-3-carboxylate (Table 4, entry 16)

^{1}H NMR (300 MHz, DMSO-d_6): δ 7.33 (t, J = 7.3 Hz, 2 H), 7.24 (d, J = 7.0 Hz, 1 H), 7.18 (d, J = 6.7 Hz, 2 H), 6.93 (s, 2 H), 4.33 (s, 1 H), 2.34 (s, 3 H), 1.04 (s, 3H) ppm.

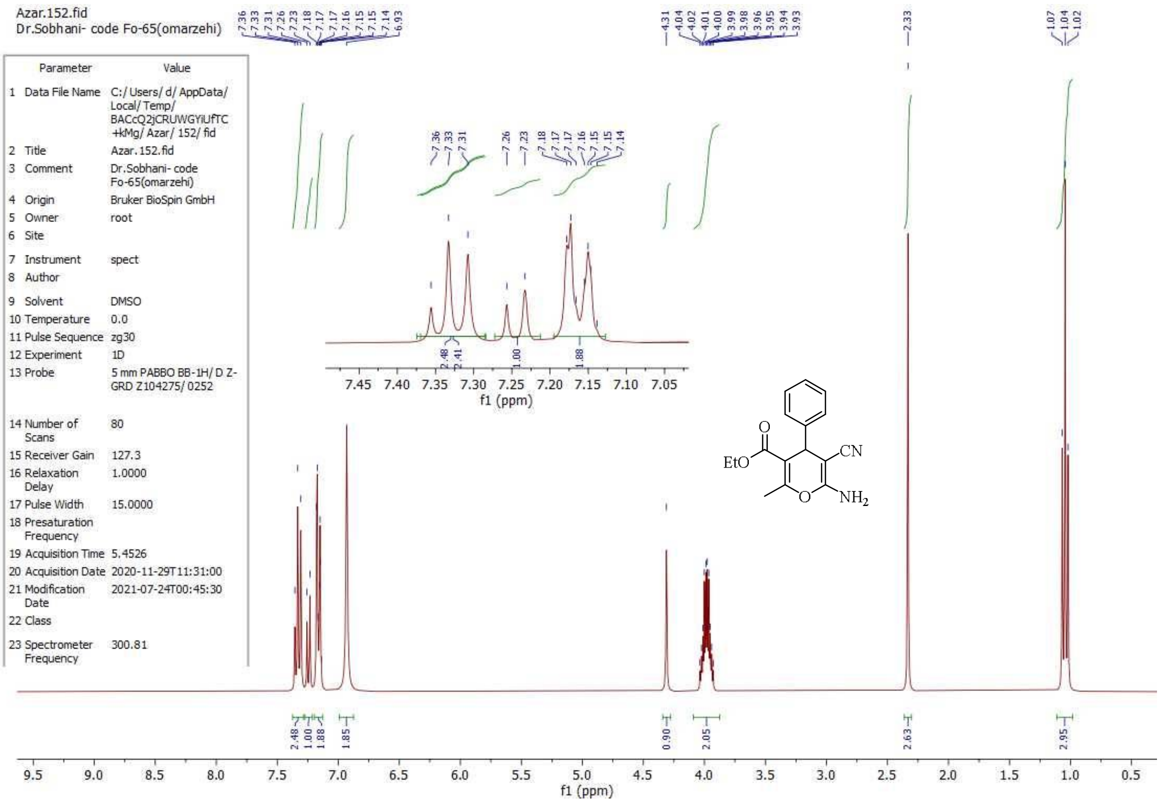

Figure S12. ^{1}H NMR spectrum of ethyl 6-amino-5-cyano-2-methyl-4-phenyl-4*H*-chromene-3-carboxylate (Table 4, entry 17)

^{1}H NMR (300 MHz, DMSO-d$_6$): δ 7.33 (t, *J* = 7.3 Hz, 2 H), 7.24 (d, *J* = 7.2 Hz, 1 H), 7.14-7.18 (m, 2 H), 6.93 (s, 2 H), 4.31 (s, 1 H), 3.93-4.04 (m, 2 H), 2.33 (s, 3 H), 1.04 (t, *J* = 7.4 Hz, 3 H) ppm.

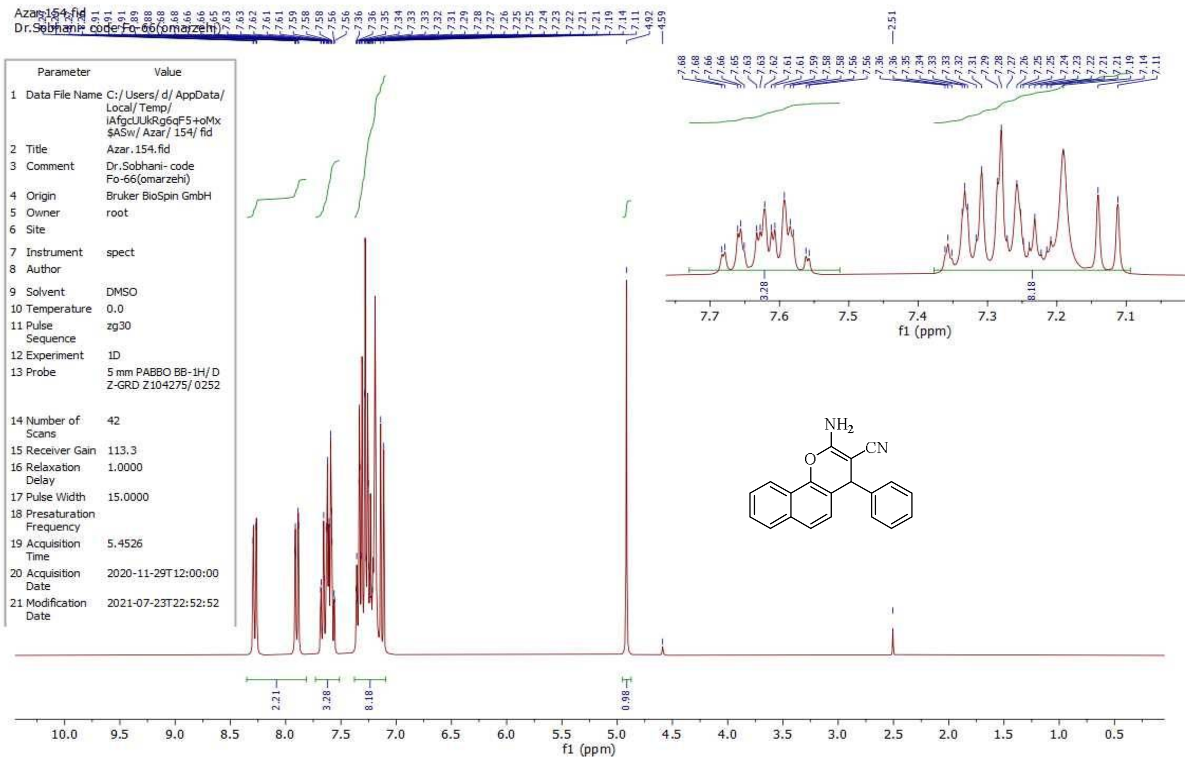

Figure S13. ^{1}H NMR spectrum of 3-amino-1-phenyl-1*H*-benzo[f]chromene-2-carbonitrile (Figure 11)

^{1}H NMR (300 MHz, DMSO-d$_6$): δ 8.21 (dd, *J* = 8.2 Hz, 1 H), 7.88-7.92 (m, 1 H), 7.53-7.73 (m, 3 H), 7.08-7.45 (m, 8 H), 4.92 (s, 1 H) ppm.

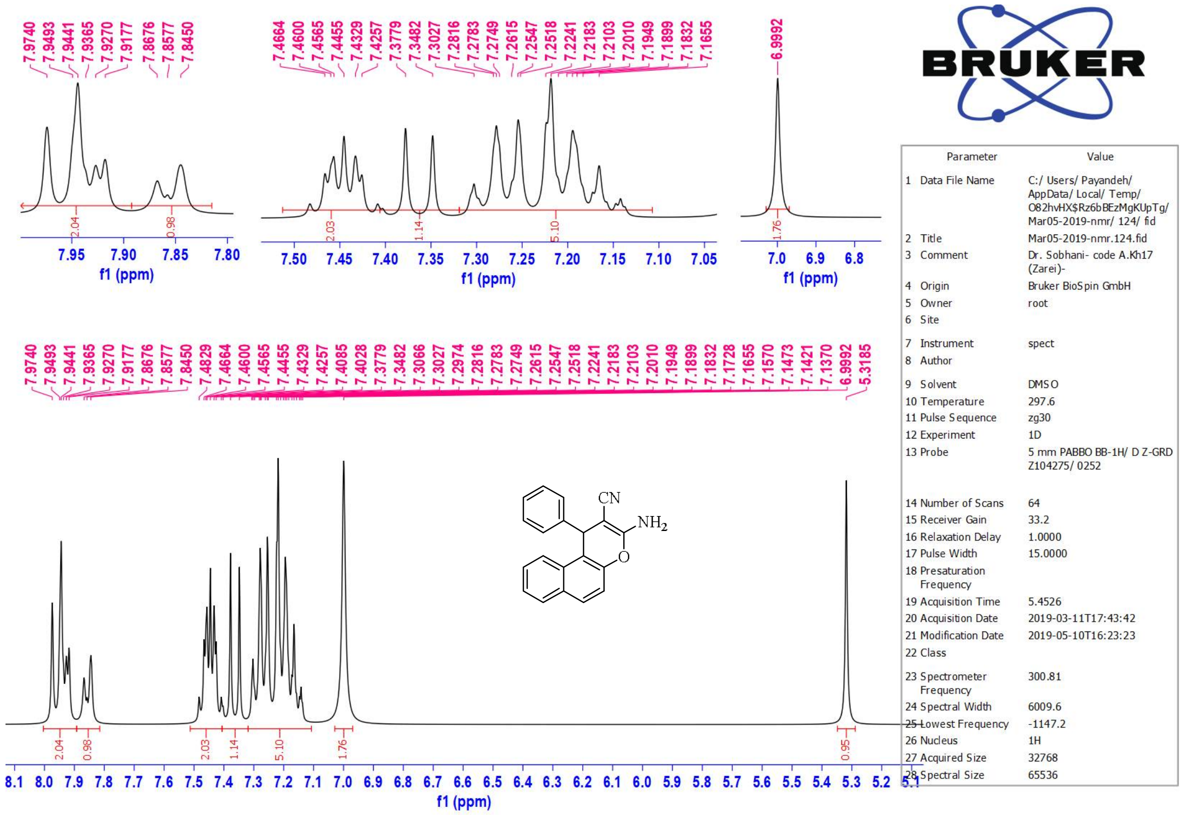

Figure S14. ^{1}H NMR spectrum of 3-amino-1-phenyl-1*H*-benzo[f]chromene-2-carbonitrile (Figure 11)

^{1}H NMR (300 MHz, DMSO-d$_6$): δ 7.84-7.97 (m, 3 H), 7.42-7.46 (m, 2 H), 7.36 (d, *J* = 8.9 Hz, 1 H), 7.16-7.28 (m, 5 H), 6.99 (s, 2 H), 5.31 (s, 1 H) ppm.

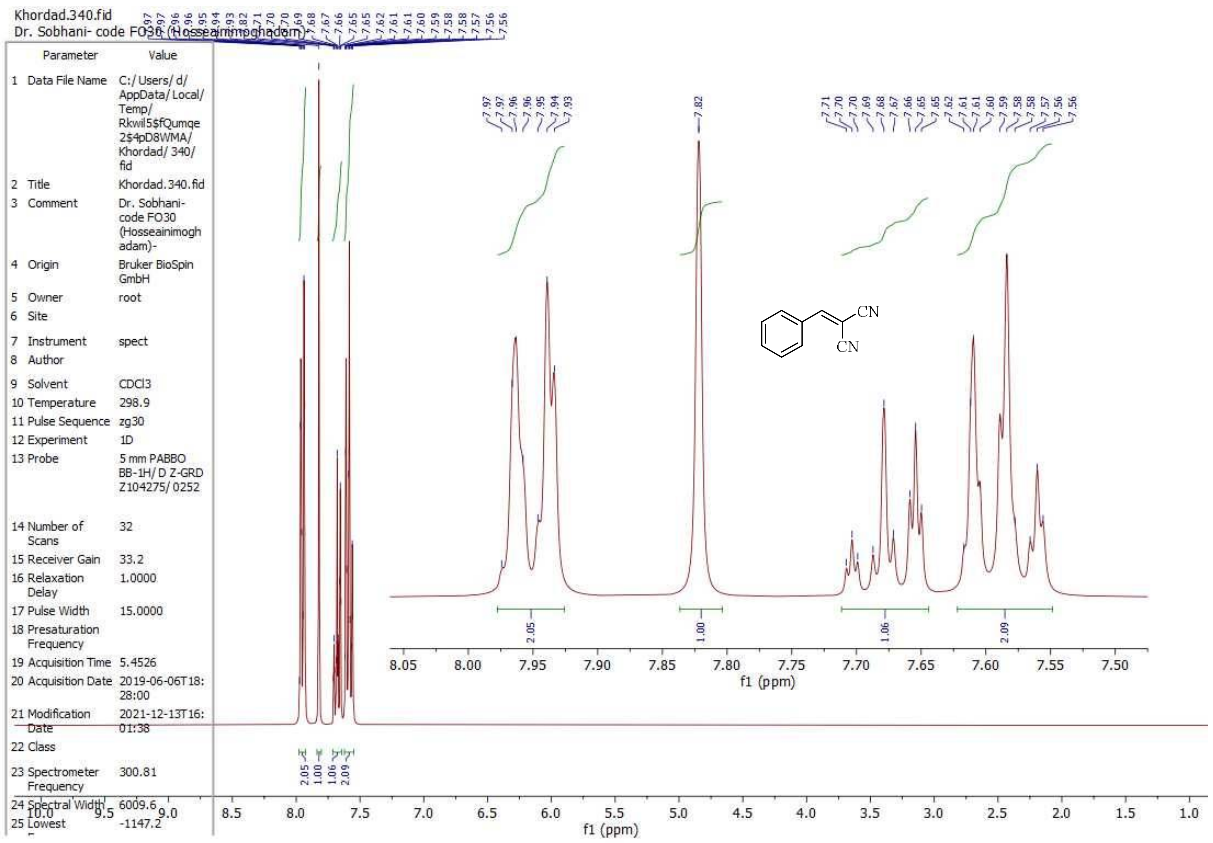

Figure S15. ^{1}H NMR spectrum of 2-benzylidene-malononitrile (Figure 16)

^{1}H NMR (300 MHz, $CDCl_3$): δ 7.93-7.97 (m, 2 H), 7.82 (s, 1 H), 7.65-7.71 (m, 1 H), 7.56-7.62 (m, 2 H) ppm.
